# Token Probabilities to Mitigate Large Language Models Overconfidence in Answering Medical Questions: Quantitative Study

**DOI:** 10.2196/64348

**Published:** 2025-08-29

**Authors:** Raphaël Bentegeac, Bastien Le Guellec, Grégory Kuchcinski, Philippe Amouyel, Aghiles Hamroun

**Affiliations:** 1 Department of Public Health, Lille University, Lille University Hospital Center, avenue du Professeur Emile Laine, Lille, 59037, France; 2 Univ. Lille, Inserm, Centre Hosp. Univ Lille, Institut Pasteur de Lille, UMR1167—Labex DISTALZ—RID-AGE—Risk Factors and Molecular Determinants of Aging-Related Diseases, Lille, France; 3 Department of Neuroradiology, Lille University, Lille University Hospital Center, Lille, France; 4 Univ. Lille, Inserm, CHU Lille, U1172—LilNCog—Lille Neuroscience & Cognition, Lille, France; 5 Univ. Lille, CNRS, Inserm, CHU Lille, Institut Pasteur de Lille, US 41 - UAR 2014 - PLBS, Lille, France

**Keywords:** ChatGPT, large language model, chatbot, confidence, token probability, natural language processing, NLP, machine learning, artificial intelligence, language model, token, probability, medical question, questionnaire, medicine, MedQA, accuracy

## Abstract

**Background:**

Chatbots have demonstrated promising capabilities in medicine, scoring passing grades for board examinations across various specialties. However, their tendency to express high levels of confidence in their responses, even when incorrect, poses a limitation to their utility in clinical settings.

**Objective:**

The aim of the study is to examine whether token probabilities outperform chatbots’ expressed confidence levels in predicting the accuracy of their responses to medical questions.

**Methods:**

In total, 9 large language models, comprising both commercial (GPT-3.5, GPT-4, and GPT-4o) and open source (Llama 3.1-8b, Llama 3.1-70b, Phi-3-Mini, Phi-3-Medium, Gemma 2-9b, and Gemma 2-27b), were prompted to respond to a set of 2522 questions from the United States Medical Licensing Examination (MedQA database). Additionally, the models rated their confidence from 0 to 100, and the token probability of each response was extracted. The models’ success rates were measured, and the predictive performances of both expressed confidence and response token probability in predicting response accuracy were evaluated using area under the receiver operating characteristic curves (AUROCs), adapted calibration error, and Brier score. Sensitivity analyses were conducted using additional questions sourced from other databases in English (MedMCQA: n=2797), Chinese (MedQA Mainland China: n=3413 and Taiwan: n=2808), and French (FrMedMCQA: n=1079), different prompting strategies, and temperature settings.

**Results:**

Overall, mean accuracy ranged from 56.5% (95% CI 54.6‐58.5) for Phi-3-Mini to 89% (95% CI 87.7‐90.2) for GPT-4o. Across the United States Medical Licensing Examination questions, all chatbots consistently expressed high levels of confidence in their responses (ranging from 90, 95% CI 90-90 for Llama 3.1-70b to 100, 95% CI 100-100 for GPT-3.5). However, expressed confidence failed to predict response accuracy (AUROC ranging from 0.52, 95% CI 0.50‐0.53 for Phi-3-Mini to 0.68, 95% CI 0.65‐0.71 for GPT-4o). In contrast, the response token probability consistently outperformed expressed confidence for predicting response accuracy (AUROCs ranging from 0.71, 95% CI 0.69‐0.73 for Phi-3-Mini to 0.87, 95% CI 0.85‐0.89 for GPT-4o; all *P*<.001). Furthermore, all models demonstrated imperfect calibration, with a general trend toward overconfidence. These findings were consistent in sensitivity analyses.

**Conclusions:**

Due to the limited capacity of chatbots to accurately evaluate their confidence when responding to medical queries, clinicians and patients should abstain from relying on their self-rated certainty. Instead, token probabilities emerge as a promising and easily accessible alternative for gauging the inner doubts of these models.

## Introduction

The potential of large language models (LLMs) for enhancing clinical workflows and facilitating communication with patients is becoming increasingly evident [1-3]. Since the public release of OpenAI’s ChatGPT in 2023, use cases in medicine have flourished, from extracting information from large volumes of documents [4] to answering questions of patients [5]. While some models are getting bigger and more capable (OpenAI’s o1, Google’s Gemini 2.0, and Anthropic’s Claude), others are focusing on data privacy and portability (Mistral Small, Meta Llama, and Microsoft Phi Mini). Their intuitive use combined with their ability to score passing grades on several board examinations including the United States Medical Licensing Examination (USMLE) is building trust in their safe deployment as medical assistants for both physicians and patients [6-10]. However, relying extensively on chatbots for health-related guidance carries the risk of misinformation and subsequent health hazards [11], as they may generate inaccurate information, hallucinate, and lack robustness [12,13]. Therefore, their use in high-stakes settings such as answering to patients’ inquiries requires a careful evaluation of both their knowledge and their ability to clearly express uncertainty [14,15].

Yet, research indicates that these models systematically express high levels of confidence in their answers, irrespective of their actual correctness [12,16]. This inability to communicate hesitations is potentially misleading for both health care professionals and patients [13,17-19]. Currently, developing a strategy to detect inner doubts of LLMs remains a major challenge for their safe use [12], as reminded directly on the ChatGPT website (“ChatGPT can make mistakes. Check important info.”). However, there currently exists no straightforward solution to detect such potential mistakes.

Interestingly, LLMs operate on a statistical model, in which each word (or token) is associated with a probability [10], which represents the model’s inner confidence in its output. Leveraging this metric in medicine could help users to identify responses that may need expert review [10,20]. Despite the potential significance of token probabilities in approximating confidence levels, research in this area remains limited [10,21]. Moreover, studies published so far have used laborious strategies, such as analyzing responses with other LLMs or answering the same question dozens of times to produce a single confidence estimate, a resource- and time-intensive strategy impractical in a clinical setting [10,22-25].

Therefore, the primary objective of this study is to evaluate and compare the predictive abilities of expressed confidence and token probabilities in the most recent LLMs—both commercial and open source—across various multilingual datasets of medical licensing examination question-answer (Q-A). We seek to establish whether token probabilities offer a more accurate method for identifying inner doubts of LLMs, ultimately enhancing the reliability of chatbot-assisted decision-making in health care settings.

## Methods

This study is reported in accordance with the STROBE (Strengthening the Reporting of Observational Studies in Epidemiology) statement.

### Models and Prompting

Models were selected to represent a diverse set of the top-performing LLMs on Measuring Massive Multitask Language Understanding in their respective size categories at the time of the study (May 2024). We selected a mix of commercial and open-source models to reflect the range of options available for practical applications. Additionally, we prioritized models with application programming interface (API) access or open weights to facilitate reproducibility. All selected models had to provide access to log probabilities in their API. As such, the Claude and Gemini models could not be used at the time of the study. Selected models were GPT-3.5, GPT-4, and GPT-4o (OpenAI), Llama 3.1-8b and Llama 3.1-70b (Meta), Phi-3-Mini and Phi-3-Medium (Microsoft), and Gemma 2-9b and Gemma 2-27b (Google). Detailed information on all the selected models is available in Table S1 in Multimedia Appendix 1. OpenAI models were accessed using the OpenAI API. All other models were accessed using Microsoft Azure’s API. We used vanilla prompting (ie, no prompt engineering), as in previous studies [12,16,26]. The vanilla prompt for all datasets and models was as follows: “Select the letter corresponding to the correct answer. In addition, please rate your confidence in your answer from 0 (not confident) to 100 (absolutely confident). Your output must follow this template, with no additional comment: ‘The correct answer is: [letter]. My confidence level is: [number].’” Temperature was set to 0 to mitigate variability [22], and a sensitivity analysis with a temperature of 0.5 was performed. Additional sensitivity analyses were performed using different prompting strategies: “expert instruction prompt” consisting of informing the model that it is a medical expert (“You are a medical expert” followed by the vanilla prompt), “confidence scaling” consisting of adjusting the requested expressed confidence on a scale from 0 to 1, and “few-shot prompting,” consisting of providing 3 examples of correct responses before the query.

Between May 29 and June 10, 2024, for the primary analysis and between December 13 and 23, 2024, for the sensitivity analyses, 9 LLMs were prompted to answer medical multiple-choice questions from datasets of medical licensing examinations in 3 languages (English, Chinese, and French; Figure S1 in Multimedia Appendix 2). These Q-A databases include validated questions and answers for medical licensing examinations in the United States (USMLE MedQA, with step 1 [basic sciences and mechanisms] and steps 2 and 3 [clinical knowledge] questions) [27], China (Mainland MCMLE MedQA) [27], Taiwan (TWMLE MedQA) [27], France (FrMedMCQA) [28], and India (MedMCQA; Table S2 in Multimedia Appendix 1) [29]. Responses were parsed using a regular expression. Answers that did not follow the template were parsed using GPT-4o.

### Confidence Measure

Two metrics were used to gauge the model’s confidence in the primary analysis: (1) the confidence expressed directly in the model’s output, as previously reported [12,16,26], called expressed confidence; and (2) the probability of the token corresponding to the model’s response (“A,” “B,” “C,” “D,” or “E”), retrieved directly from the API, called response token probability. Token probabilities (Figure S1 in Multimedia Appendix 2) are the likelihood of each token to appear at a given point during the generation process, contingent on the preceding context and model parameters [30]. In a Q-A context reliant on token values, token probabilities have been leveraged to implicitly evaluate labeling certainty [20]. Response token probabilities were not normalized to assess the simplest measure available for end users.

In secondary analyses, two additional metrics were evaluated: (1) the Shannon entropy of the response token distribution, a measure of the dispersion of the probabilities of possible response tokens [31]; and (2) perplexity, a measure of the overall probability of the answer generated by the model, defined as the product of one over the probability of each token that appeared in the response [31].

### Statistical Analysis

Accuracy estimates, along with their 95% CIs, were compared across the 7 LLMs within each Q-A dataset using the Fisher exact test. The means and distribution of expressed confidence, token probability, Shannon entropy, and answer perplexity were visually represented using violin plots and compared based on response correctness. Predictive performance was assessed through receiver operating characteristic curves, presenting area under the receiver operating characteristic curve (AUROC) with 95% CIs, and compared between expressed confidence and response token probabilities using the DeLong test. Optimal discrimination thresholds were determined using the Youden *J* statistic. True positive rate, true negative rate, as well as accuracy rates above and below optimal discrimination threshold were estimated with 95% CIs and compared using McNemar tests. Model confidence calibration was evaluated using calibration curves and quantified based on both the adaptive calibration error (ACE), a measure of the average deviation across confidence bins, using adaptive binning to mitigate dataset imbalances, and Brier score, a measure of both accuracy and calibration by computing the mean squared difference between predicted probabilities and actual outcomes, with their respective 95% CI (bootstrap) [32,33], both metrics were compared using bootstrapped *P* values. ACE greater than 0.25 indicates poor calibration. All statistical analyses were performed using R (version 4.4; R Foundation for Statistical Computing) and Python (version 3.12; Python Software Foundation), with a significance threshold set at 5%.

### Ethical Considerations

All data used for this study are publicly available and do not involve patient data. As such, no ethics review board assessment was necessary.

## Results

### Overall Performance

GPT-4o significantly outperforms all other tested models with an accuracy of 89% (95% CI 87.7‐90.2; *P*<.001; Table S3 in Multimedia Appendix 1 and Figure S2 in Multimedia Appendix 2). Phi-3-Mini, the smallest model tested, scores the lowest score of 56.5% (95% CI 54.6‐58.5). Considering a passing grade of 60%, GPT-3.5, GPT-4, GPT-4o, Llama 3.1-70b, Phi-3-Medium, and Gemma 2-27b pass the USMLE board with vanilla prompting. No model scores an “expert grade” of more than 90%. Similar performance patterns are visible with the other datasets, with larger models scoring higher than their smaller counterparts (Figure S2 in Multimedia Appendix 2).

Different prompting strategies and temperatures had limited effect on the performance of tested models, except for GPT-3.5 Turbo, which performed significantly better on MedQA with few-shot prompting (accuracy of 63.5, 95% CI 61.6‐65.4 vs 60.1, 95% CI 58.2‐62.0; *P*=.01; Table S4 in Multimedia Appendix 1 and Figure S3 in Multimedia Appendix 2).

### Uncertainty Quantification

When elicited to assess their confidence in their answer, all models verbalized high scores, notably higher than 80%, in multiples of 5, and with limited dispersion around the median value. Expressed confidence ranged from 90 (95% CI 90-90) for Llama 3.1-70b to 100 (95% CI 100-100) for GPT-3.5. All models consistently expressed a confidence level exceeding 80/100 in their responses, regardless of the accuracy of the answer, particularly for GPT-3.5, GPT-4, Phi-3-Mini, and Phi-3-Medium (Table S3 in Multimedia Appendix 1).

**Figure 1.**
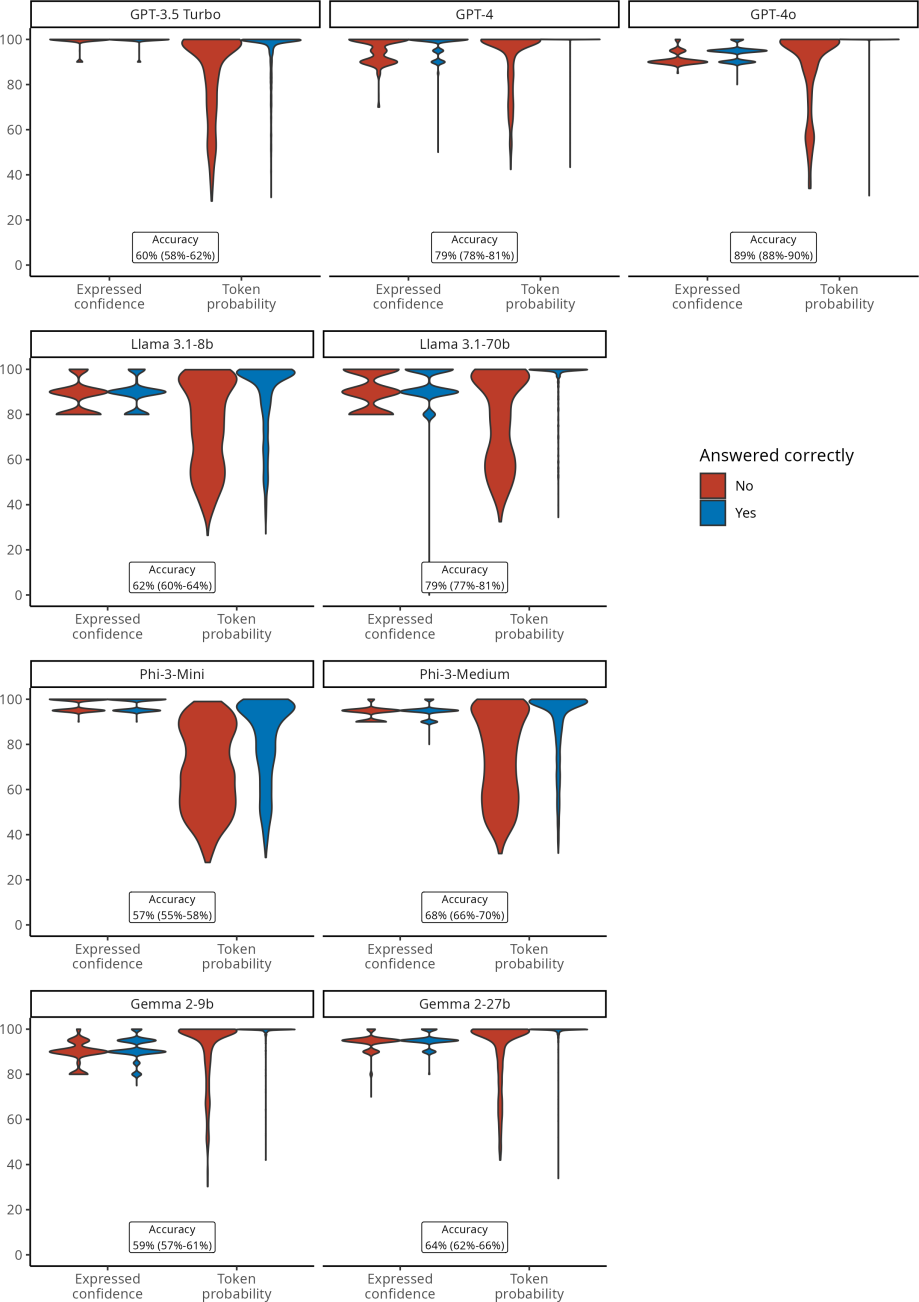
Distributions of expressed confidence versus token probability of large language models on the US MedQA dataset (n=2487), according to the accuracy of model answers.

Response token probabilities were also skewed toward high values for all models but displayed more dispersed values, ranging from 93 (95% CI 71-99) for Phi-3-Medium to 100 (95% CI 100-100) for GPT-4 (Figure 1). Response token probabilities were significantly more likely to be above 80/100 for correct answers than for incorrect ones, across all models (all *P*<.001). As illustrated in Figure 1, the qualitative analysis of response token probabilities revealed a frequent dispersion of probabilities among the options when the chatbot’s response is incorrect. Contrarily, when the model’s chosen response was correct, the token probability distribution is often almost entirely directed toward a single option (Figure 1).

The analyses replicated on other Q-A datasets confirmed the models’ tendency to express high levels of confidence (Tables S5-S8 in Multimedia Appendix 1 and Figures S2-S5 in Multimedia Appendix 2).

### Discriminative Power of Expressed Confidence Versus Response Token Probability

**Figure 2.**
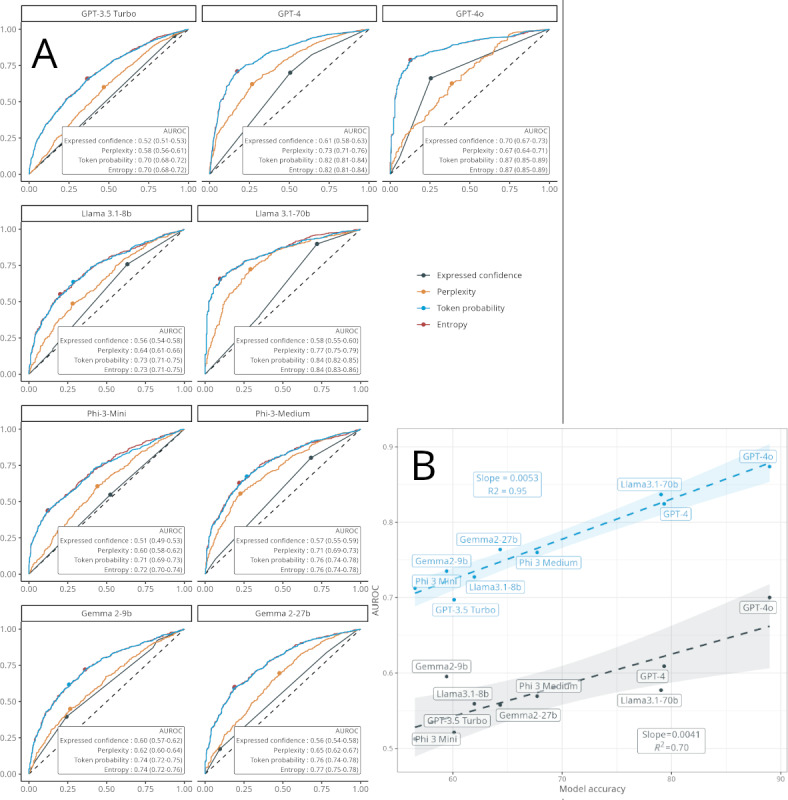
(A) Receiver operating characteristic curve and discriminative power of expressed confidence versus perplexity, entropy, and token response probability in predicting answer accuracy of large language models. (B) Plot of the AUROC of expressed confidence and response token probability as a function of models’ accuracy on the US MedQA dataset (n=2487 questions). AUROC: area under the receiver operating characteristic curve.

Overall, expressed confidence failed to predict the accuracy of responses, whereas response token probabilities demonstrated satisfactory to very good discriminatory performance. The AUROCs of expressed confidence ranged from 0.51 (95% CI 0.49‐0.53) for Phi-3-Mini to 0.70 (95% CI 0.67‐0.73) for GPT-4o (Figure 2A and Table 1). In all cases, response token probability markedly outperformed expressed confidence (all *P*<.001), with AUROCs ranging from 0.71 (95% CI 0.69‐0.73) for Phi-3-Mini to 0.87 (95% CI 0.85‐0.89) for GPT-4o (Figure 2A and Table 1).

**Table 1.** Diagnostic performance accuracy of expressed confidence versus token response probability, according to the different models (US MedQA; n=2522 questions).

Model and metric		Expressed confidence	Token probability	*P* value
GPT-3.5 Turbo				
	AUROC^a^ (95% CI)	0.52 (0.51‐0.53)	0.7 (0.68‐0.72)	<.001
	Optimal threshold (%)	98	98	—^b^
	True positive rate (%) (95% CI)	95 (94‐96)	66 (64‐68)	<.001
	False positive rate (%) (95% CI)	91 (89‐93)	37 (34‐40)	<.001
	Correct above threshold (%) (95% CI)	61 (59‐63)	73 (71‐75)	<.001
	Correct below threshold (%) (95% CI)	44 (36‐52)	45 (42‐48)	.84
GPT-4				
	AUROC (95% CI)	0.61 (0.58‐0.63)	0.82 (0.81‐0.84)	<.001
	Optimal threshold (%)	98	100	—
	True positive rate (%) (95% CI)	70 (68‐72)	71 (69‐73)	.44
	False positive rate (%) (95% CI)	51 (46‐55)	17 (14‐21)	<.001
	Correct above threshold (%) (95% CI)	84 (82‐86)	94 (93‐95)	<.001
	Correct below threshold (%) (95% CI)	70 (67‐73)	57 (54‐60)	<.001
GPT-4o				
	AUROC (95% CI)	0.7 (0.67‐0.73)	0.87 (0.85‐0.89)	<.001
	Optimal threshold (%)	92	100	—
	True positive rate (%) (95% CI)	66 (64‐68)	79 (77‐81)	<.001
	False positive rate (%) (95% CI)	26 (20‐31)	13 (8.8‐17)	<.001
	Correct above threshold (%) (95% CI)	95 (94‐96)	98 (97‐99)	<.001
	Correct below threshold (%) (95% CI)	79 (76‐81)	66 (63‐70)	<.001
Llama 3.1-8b				
	AUROC (95% CI)	0.56 (0.54‐0.58)	0.73 (0.71‐0.75)	<.001
	Optimal threshold (%)	85	91	—
	True positive rate (%) (95% CI)	76 (74‐78)	64 (61‐66)	<.001
	False positive rate (%) (95% CI)	63 (60‐66)	29 (26‐31)	<.001
	Correct above threshold (%) (95% CI)	66 (64‐68)	78 (76‐81)	<.001
	Correct below threshold (%) (95% CI)	52 (48‐55)	45 (43‐48)	<.001
Llama 3.1-70b				
	AUROC (95% CI)	0.58 (0.55‐0.6)	0.84 (0.82‐0.85)	<.001
	Optimal threshold (%)	85	99	—
	True positive rate (%) (95% CI)	90 (89‐91)	66 (63‐68)	<.001
	False positive rate (%) (95% CI)	72 (68‐76)	9.6 (7.1‐12)	<.001
	Correct above threshold (%) (95% CI)	82 (81‐84)	96 (95‐97)	<.001
	Correct below threshold (%) (95% CI)	58 (52‐63)	59 (56‐62)	.57
Phi-3-Mini				
	AUROC (95% CI)	0.51 (0.49‐0.53)	0.71 (0.69‐0.73)	<.001
	Optimal threshold (%)	98	92	—
	True positive rate (%) (95% CI)	55 (52‐57)	44 (42‐47)	<.001
	False positive rate (%) (95% CI)	52 (49‐55)	12 (10‐14)	<.001
	Correct above threshold (%) (95% CI)	58 (55‐60)	82 (80‐85)	<.001
	Correct below threshold (%) (95% CI)	55 (52‐58)	45 (43‐48)	<.001
Phi-3-Medium				
	AUROC (95% CI)	0.57 (0.55‐0.59)	0.76 (0.74‐0.78)	<.001
	Optimal threshold (%)	92	91	—
	True positive rate (%) (95% CI)	80 (78‐82)	67 (65‐70)	<.001
	False positive rate (%) (95% CI)	68 (65‐71)	27 (24‐30)	<.001
	Correct above threshold (%) (95% CI)	71 (69‐73)	84 (82‐86)	<.001
	Correct below threshold (%) (95% CI)	56 (52‐60)	48 (45‐51)	<.001
Gemma 2-9b				
	AUROC (95% CI)	0.6 (0.57‐0.62)	0.74 (0.72‐0.75)	<.001
	Optimal threshold (%)	92	100	—
	True positive rate (%) (95% CI)	40 (37‐42)	62 (59‐64)	<.001
	False positive rate (%) (95% CI)	24 (22‐27)	26 (23‐28)	.40
	Correct above threshold (%) (95% CI)	71 (67‐74)	78 (75‐80)	<.001
	Correct below threshold (%) (95% CI)	54 (51‐56)	43 (40‐46)	<.001
Gemma 2-27b				
	AUROC (95% CI)	0.56 (0.54‐0.58)	0.76 (0.74‐0.78)	<.001
	Optimal threshold (%)	98	100	—
	True positive rate (%) (95% CI)	17 (15‐19)	60 (57‐62)	<.001
	False positive rate (%) (95% CI)	9.5 (7.5‐11)	19 (16‐21)	<.001
	Correct above threshold (%) (95% CI)	77 (72‐81)	85 (83‐87)	<.001
	Correct below threshold (*%)* (95% CI)	62 (60‐64)	47 (45‐50)	<.001

a AUROC: area under the receiver operating characteristic curve.

b Not available.

There was a positive linear correlation between both expressed confidence and response token probability and the models’ accuracy, with the discriminative power of both metrics increasing with the accuracy of the model (Figure 2B).

The sensitivity analyses conducted on the other multilingual Q-A datasets confirmed the consistent superior discriminative capabilities of response token probability in predicting response accuracy compared to expressed confidence (Tables S5-S8 in Multimedia Appendix 1 and Figures S4-S11 in Multimedia Appendix 2). Slightly lower performance of the response token probability was observed on the MedQA Mainland China dataset for smaller models, with AUROCs for Phi-3-Mini at 0.56 (95% CI 0.54‐0.59) and Llama 3.1-8b at 0.59 (95% CI 0.57‐0.62).

### Predictive Performance of Expressed Confidence Versus Response Token Probability

Threshold values to optimize the models’ discriminative abilities to predict accuracy were high for both expressed confidence and response token probability. These values ranged from 85% (Llama 3.1-8b and Llama 3.1-70b) to 98% (GPT-3.5 Turbo, GPT4, Phi-3-Mini, and Gemma 2-27b) for expressed confidence and from 91% (Llama 3.1-8b and Phi-3-Medium) to 100% (GPT-4, GPT-4o, Gemma 2-9b, and Gemma 2-27b) for token probability (Table 1).

All models exhibited significantly higher accuracy when the response token probability exceeded its optimal threshold, ranging from 73% (95% CI 71-75) for GPT-3.5 Turbo to 98% (95% CI 97-99) for GPT-4o (all *P*<.001). In comparison, when expressed confidence was at its respective optimal threshold, accuracy ranged from 58% (95% CI 55-60) for Phi-3-Mini to 95% (95% CI 94-96) for GPT-4o (all *P*<.001; Table 1).

Moreover, the false positive rate (incorrect responses above threshold) was significantly lower for answers with high response token probability, ranging from 9.6% (95% CI 7.1‐12) for Llama 3.1-70b to 37% (95% CI 34-40) for GPT-3.5 Turbo (all *P*<.001). In contrast, for answers with high expressed confidence, the false positive rates ranged from 9.5% (95% CI 7.5‐11) for Gemma 2-27b to 91% (95% CI 89-93) for GPT-3.5 Turbo (all *P*<.001 except Gemma 2-9b, *P*=.40; Table 1). Similar results are observed across all other Q-A datasets (Tables S5-S8 in Multimedia Appendix 1).

### Calibration Error

**Figure 3.**
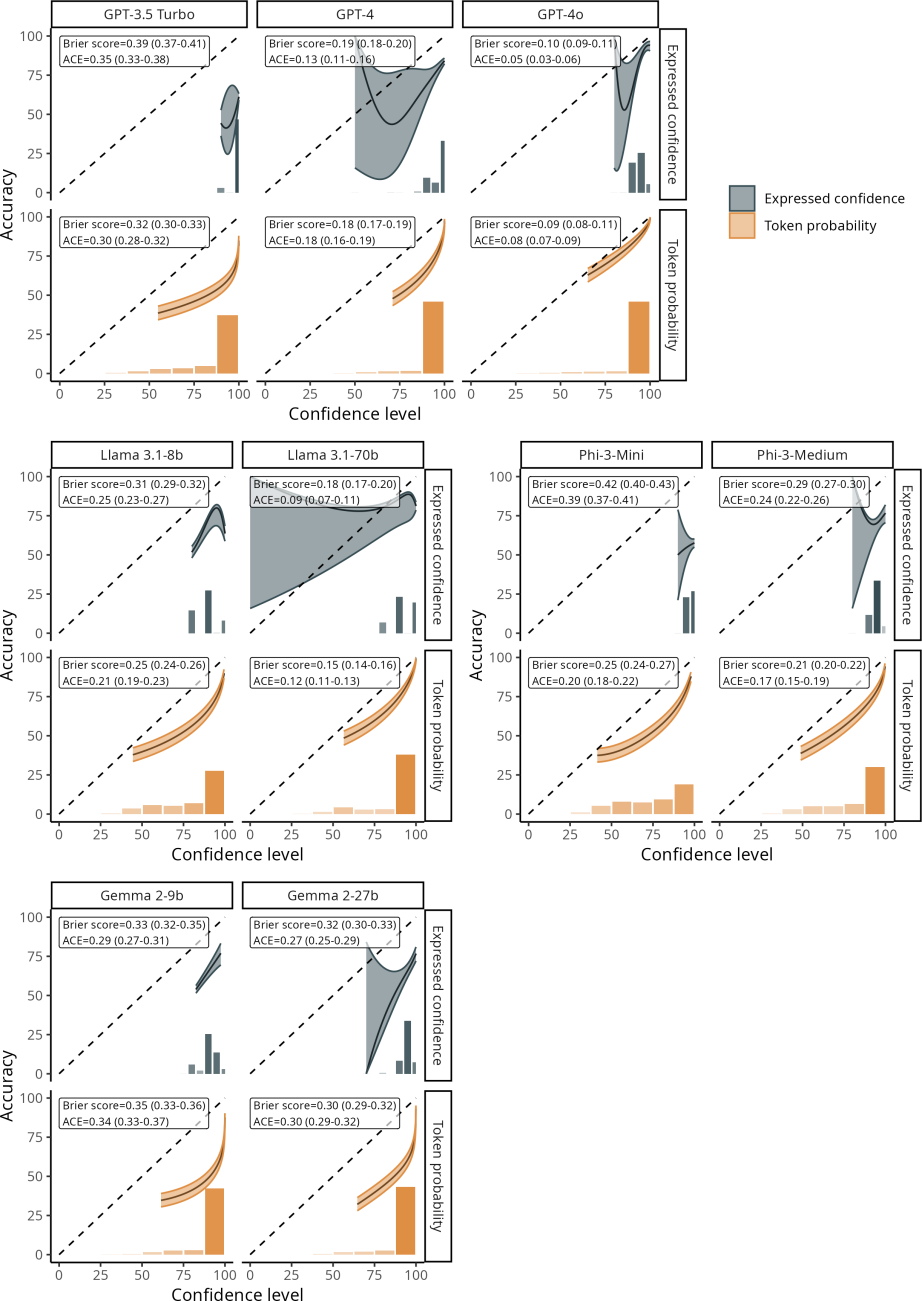
Calibration plots comparing expressed confidence versus token response probability in predicting answer accuracy of large language models (US MedQA dataset: n=2487 questions). ACE: adapted calibration error.

All models exhibited a tendency toward overconfidence, with smaller models being the more poorly calibrated (Figure 3). ACE ranged from over 20%‐25% for Phi-3-Medium and Llama 3.1-8b to over 30% for GPT-3.5 and even exceeding 40% for Phi-3-Mini (Figure 3). In contrast, the larger models demonstrated satisfactory calibration for both expressed confidence and response token probability, notably less than 10% with GPT-4o. Overall, all models exhibited significantly lower Brier scores, meaning better calibration with response token probability, ranging from 0.09 (95% CI 0.08‐0.11) for GPT-4o to 0.35 (95% CI 0.33‐0.36) for Gemma 2-9b, than with expressed confidence, ranging from 0.10 (95% CI 0.09‐0.11) for GPT-4o to 0.42 (95% CI 0.40‐0.43) for Phi-3-Mini (all *P*<.05; Figure 3). Similarly, ACEs were almost always lower with response token probability than with expressed confidence, although the larger models sometimes showed lower ACEs on expressed confidence. The same tendency toward overconfidence is highlighted across other Q-A datasets, with poor calibration observed for smaller models and a significantly lower Brier score with response token probability (Tables S9-S13 in Multimedia Appendix 1 and Figures S15-S20 in Multimedia Appendix 2).

### Effect of Knowledge Type

Response token probability generally performed similarly across step 1 and steps 2 and 3 items from MedQA for most models, with no significant differences observed between these knowledge categories. In particular, GPT-4 showed area under the curves (AUCs) of 0.80 (95% CI 0.82‐0.85) versus 0.79 (95% CI 0.82‐0.85; *P*=.92), GPT-4o had 0.84 (95% CI 0.87‐0.90) versus 0.85 (95% CI 0.88‐0.91; *P*=.68), and Llama 3.1-70b demonstrated 0.81 (95% CI 0.84‐0.86) versus 0.81 (95% CI 0.84‐0.86; *P*=.99). Similarly, GPT-3.5 Turbo, Llama 3.1-8b, Phi 3, and Gemma 2 models all displayed minimal AUC differences between basic sciences and clinical knowledge (all *P*>.05; Tables S14-S16 in Multimedia Appendix 1).

### Effect of Prompting Strategies

**Figure 4.**
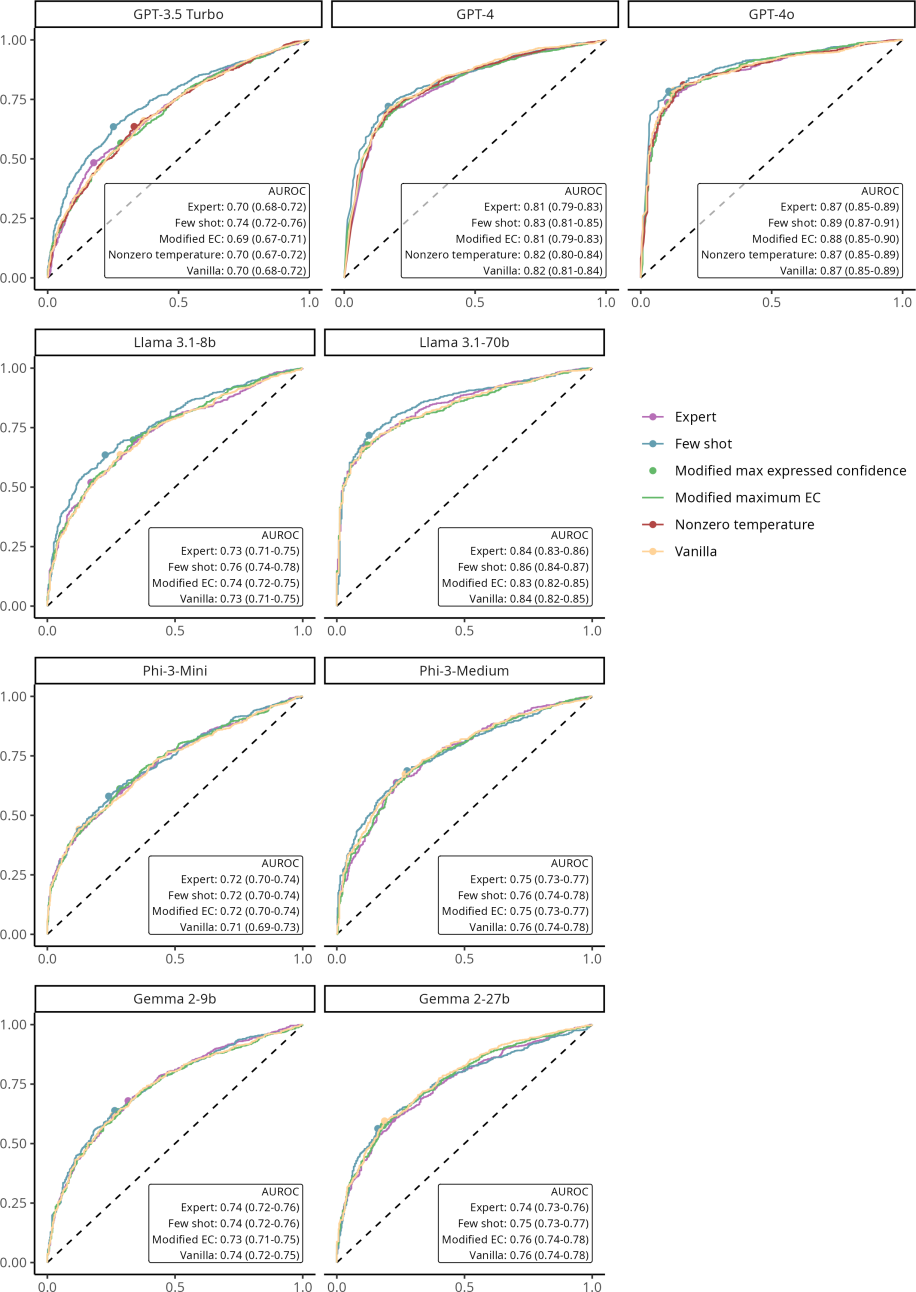
Effect of prompting techniques on receiver operating characteristic curve and discriminative power of the response tokens’ probability in predicting answer accuracy of large language models (US MedQA dataset: n=2487 questions). AUROC: area under the receiver operating characteristic curve; EC: expressed confidence.

Vanilla prompts generally performed on par with other prompting strategies for most models, with only small differences with the few-shot prompt. In particular, GPT-3.5 Turbo and Llama 3.1-8b showed significant advantages for few shot over vanilla (AUC increases from 0.70 to 0.74; *P*=.002 and 0.73 to 0.76; *P*=.01, respectively), while the remaining comparisons in GPT-4, GPT-4o, Llama 3.1-70b, Phi 3, and Gemma 2 models did not detect statistically meaningful deviations from vanilla (Figure 4 and Table S17 in Multimedia Appendix 1).

### Shannon Entropy and Answer Perplexity

Shannon entropy achieved performance comparable to token‐level probabilities across nearly all models, typically showing overlapping AUC estimates and minimal statistical deviations. For example, with GPT-4, the entropy AUC was 0.82 (95% CI 0.80‐0.84) versus 0.82 (95% CI 0.81‐0.84) for token probability (*P*=.41), and GPT-4o yielded 0.87 (95% CI 0.85‐0.89) for both metrics (*P*=.16). By contrast, perplexity consistently underperformed relative to token probability in all models, as exemplified by Llama 3.1-70b, which had an AUC of 0.77 (95% CI 0.75‐0.79) for perplexity and 0.84 (95% CI 0.83‐0.86) for token probability (*P*<.001; Table S18 in Multimedia Appendix 1 and Figure 2A).

## Discussion

### Principal Findings

This study is the first attempt to try to control the risk of misinformation in the use of medical chatbots by comparing the predictive value of expressed confidence and response token probabilities for various LLMs when addressing medical questions from diverse multilingual medical licensing examination datasets. The findings demonstrate the robust performance of LLMs in this domain, displaying an accuracy rate for USMLE ranging from approximately 60% for smaller models (GPT-3.5, Llama 3-8b, and Phi-3-Mini) to nearly 90% for GPT-4o, and mostly succeeding in medical board examinations across different languages. A slight drop in performance was observed for the smallest models on the Mainland China dataset, probably because of the underrepresentation of Chinese texts in the training data of these models. Notably, different prompting strategies had little to no impact on the performance of response token probabilities across all datasets and models tested. Furthermore, the results underscore a consistent tendency among LLMs to exhibit high expressed confidence in their responses, with scores typically ranging between 80% and 100%, regardless of their actual accuracy in most cases. While response token probabilities display a similar inclination due to their imperfect calibration, they markedly outperform expressed confidence in predicting response accuracy, with very good predictive abilities among the larger models.

### Limitations

This study, however, has several limitations. First, we restricted our analysis to single-choice questions. Consequently, these findings may not be directly applicable to textual responses, for which the model’s decision is not confined to a single token. While there are proposed methods for aggregating token probabilities, they are still under development [10,23]. Second, we could only assess the effect of some variations of prompt-engineering techniques as well as 2 temperature settings, while other techniques and parameters could have potentially impacted the outcomes. However, we deliberately opted for using LLMs in their default configuration to mimic real-world use by standard users, as in previous reports [3,12]. Finally, our study did not exhaustively cover all available LLMs and languages. However, by integrating various chatbots of different sizes and architectures, both commercial and open source, we aimed to encompass a broad spectrum of models currently in use.

### Comparison With Prior Work

Our findings on the performance of LLMs in achieving passing grades on board-style examinations align with existing literature. Initial studies demonstrated GPT-3.5’s ability to score over 60% on the USMLE less than 2 years ago [6]. Since then, both commercial and open-source models have improved their scores through vanilla prompting and prompt engineering. During this period, models have become larger and more capable, with GPT-4o achieving nearly expert-level grades. Simultaneously, lighter models, such as Llama 3.1-70b or Phi 3, which are at least 10 times smaller than GPT-3.5, are now also capable of scoring passing grades on the USMLE.

Despite the impressive performance of LLMs on medical benchmarks, our research supports previous findings regarding their tendency to consistently overestimate their confidence levels (see literature review in Table S19 in Multimedia Appendix 1) [10,12,25,34-36]. Language models typically assess their confidence between 80 and 100, often in increments of 5, potentially imitating human patterns of expressing confidence [26]. This tendency is even more obvious in smaller models, where confidence levels remain high regardless of response accuracy. In a recent study by Krishna et al [12], ChatGPT-3.5 and 4 were asked to answer 150 radiology board-style multiple-choice text-based questions and rate their confidence level from 1 to 10. Both models consistently estimated their confidence level at 8/10 or higher, even when the response was incorrect (in 100% of cases for ChatGPT-3.5 and 77% for ChatGPT-4) [12]. One underlying explanation for this overconfidence is the exposure of LLMs to exaggerated expressions in training data, where numbers are commonly used in a figurative manner [13]. Recently, Farquhar et al [23] have also explored probabilistic approaches to detect hallucinations in LLMs’ responses. However, their method relies on a comprehensive analysis of all answering possibilities for every query, which implies significant computational complexity and costs. Such a method is not compatible with commercial models run on the cloud such as ChatGPT, and as such, seems currently impractical in a clinical setting. Additionally, their study did not include medical questions and languages other than English, leaving an easily implementable method for patients and physicians still to be explored [23].

Distinguishing when language models know and when they hesitate is crucial in high-stakes settings such as decision-making in medicine. Kung et al [37] demonstrated that the density of insight in GPT-3.5’s responses to the USMLE was lower when the model provided incorrect answers. However, only an expert can accurately assess the actual amount of knowledge contained in a language model’s output. We hypothesize that this lack of insight could manifest as a lower probability in the answer token. When the model knows the answer, it outputs a token with near-certain probability. Conversely, when it doubts because of a lack of insight, the probabilities are distributed across multiple possible answers, lowering the response token probability. Our results showing a correlation between models’ accuracy and predictive value of both expressed confidence and response token probability further strengthen this hypothesis. Higher-performing models have learned more robust internal representations, thus allowing them to generate more meaningful token probabilities as well as identify what they do not know.

LLMs demonstrate high performance in medical tasks [37], suggesting their imminent integration into patient daily lives and physician workflows. Assessing the risk of incorrect answers would further increase the trustworthiness of those emerging assistants by identifying potentially doubtful answers that could need human review. Because response token probabilities do not need advanced prompting techniques, they could be easily adopted by the medical community as a way to implicitly measure the ability of models to express their doubts. When LLMs are deployed for labeling large amounts of data, be it for extracting information from free text or correcting errors in medical reports, response token probabilities could pinpoint cases necessitating expert review, thereby reducing error risk, human effort, and financial costs [4,38].

### Future Directions

Future work should focus on investigating uncertainty estimation methods in contexts beyond closed-form, single-token responses, such as open-ended reasoning tasks or multistep problem-solving. With the release of reasoning models, integrating Chain-of-Thought architectures will also pose greater challenges for uncertainty estimation, as their decision process involves multiple reasoning steps rather than a single-token prediction. Additionally, assessing the impact of fine-tuning models on medical-specific corpora to determine whether expressed confidence calibration can be improved. Finally, future work will need to explore the practical implementation of uncertainty estimation methods in real-world settings, particularly in clinical applications. This includes evaluating how these measures can be effectively integrated into medical decision-making workflows, ensuring that they are interpretable and actionable for health care professionals, and designing interfaces that facilitate their adoption in high-stakes environments.

### Conclusions

Our study underscores the robust performance of language models in addressing medical questions across diverse multilingual databases. Token probabilities emerge as a promising alternative for predicting response accuracy, counterbalancing a consistent tendency toward overconfidence, warranting further investigation for enhanced model confidence estimation.

## Supplementary material

10.2196/64348 Multimedia Appendix 1 Additional tables.

10.2196/64348 Multimedia Appendix 2 Additional figures.

## Abbreviations

ACE: adaptive calibration error
API: application programming interface
AUC: area under the curve
AUROC: area under the receiver operating characteristic curve
LLM: large language model
Q-A: question-answer
STROBE: Strengthening the Reporting of Observational Studies in Epidemiology
USMLE: United States Medical Licensing Examination
