# Supplementary material for: Token Probabilities to Mitigate Large Language Models Overconfidence in Answering Medical Questions: Quantitative Study
Source: J Med Internet Res. 2025 Aug 29;27:e64348. doi: 10.2196/64348 (PMC12396779; doi:10.2196/64348)
Supplement: Multimedia Appendix 1 [file jmir-v27-e64348-s001.docx]

**Table S1.** Characteristics of the Large Language Models.

| **Model** | **Creator** | **License** | **Version** | **Release date** | **Parameter count** | **MMLU** |
| --- | --- | --- | --- | --- | --- | --- |
| **GPT-3.5 Turbo** | OpenAI | Commercial | 0125 | 2024-01-25 | Unknown | 70.0* |
| **GPT-4** | OpenAI | Commercial | 0613 | 2023-06-13 | Unknown | 86.4* |
| **GPT-4o** | OpenAI | Commercial | 2024-11-20 | 2024-11-20 | Unknown | 88.7* |
| **Llama3.1-8b** | Meta | Open weights | Instruct | 2024-07-23 | 8 billion | 69.4 |
| **Llama3.1-70b** | Meta | Open weights | Instruct | 2024-07-23 | 70 billion | 83.6 |
| **Phi 3 Mini** | Microsoft | Open weights | 4k-Instruct | 2024-06-27 | 3.8 billion | 70.9 |
| **Phi 3 Medium** | Microsoft | Open weights | 4k-Instruct | 2024-05-21 | 14 billion | 78.0 |
| **Gemma2-9b** | Google | Open weights | Instruct | 2024-06-27 | 9 billion | 71.3 |
| **Gemma2-27b** | Google | Open weights | Instruct | 2024-06-27 | 27 billion | 75.2 |

*MMLU of closest model

**Table S2**. Characteristics of the Medical Board Examination Questions-Answer Datasets.

|  | **MedQA USMLE** | **MedQA TWMLE** | **MedQA MCMLE** | **MedMCQA** | **French MedMCQA** |
| --- | --- | --- | --- | --- | --- |
| **Country (year)** | USA (2020) | Taiwan (2020) | China (2020) | India (2022) | France (2022) |
| **Language** | English | Traditional Chinese | Simplified Chinese | English | French |
| **Number of questions** | 2,487 | 2,734 | 3,414 | 2,763 | 1,076 |
| **Answer options** | 4-option: A/B/C/D  Only one answer | 4-option: A/B/C/D  Only one answer | 4-option: A/B/C/D  Only one answer | 5-option: A/B/C/D/E  Only one answer | 5-option: A/B/C/D/E  Only one answer |
| **Source** | National medical board examination | National medical board examination | National medical board examination | National-level postgraduate medical entrance exam | National-level medical board examination in Pharmacy |
| **Topics** | Medical knowledge, clinical problem-solving | Medical knowledge, clinical problem-solving | Medical knowledge, clinical problem-solving | Diagnostic Reasoning and Treatment, Pharmacology, Psychology, Biology, Physical Examination, General Management Strategies, Medical Knowledge | Real-world medical exam questions (Medicine, Surgery, Radiology, and Biochemistry) |
| **Passing grade** | 60% | 60% | 60% | 50% | 50% |

**Table S3**. Accuracy, expressed confidence and token response probability according to different models (USMedQA dataset, n = 2,487 questions).

| **Model** | **Accuracy** | **Expressed confidence** *median (Q1, Q3)* | **Token probability** *median (Q1, Q3)* | **Expressed confidence > 80/100**  *(correct versus incorrect answers)** | **Token probability > 80/100**  *(correct versus incorrect answers)** |
| --- | --- | --- | --- | --- | --- |
| **GPT-3.5 Turbo** | 60.1 (58.2 - 62.0) | 100 (100 - 100) | 98.7 (86.7 - 99.8) | 100% vs 100% P>.99 | 88% vs 70% P<.001 |
| **GPT-4** | 79.3 (77.7 - 80.9) | 100 (95 - 100) | 100.0 (99.9 - 100.0) | 100% vs 99% P=.001 | 97% vs 82% P<.001 |
| **GPT-4o** | 89.0 (87.7 - 90.2) | 95 (90 - 95) | 100.0 (99.9 - 100.0) | 100% vs 100% P>.99 | 96% vs 77% P<.001 |
| **Llama3.1-70b** | 79.1 (77.4 - 80.6) | 90 (90 - 100) | 99.4 (89.1 - 99.9) | 90% vs 72% P<.001 | 88% vs 51% P<.001 |
| **Llama3.1-8b** | 62.0 (60.0 - 63.9) | 90 (80 - 90) | 90.9 (69.0 - 98.5) | 76% vs 63% P<.001 | 77% vs 45% P<.001 |
| **Phi 3 Medium** | 67.7 (65.8 - 69.5) | 95 (95 - 95) | 93.5 (72.3 - 99.1) | 100% vs 100% P>.99 | 80% vs 45% P<.001 |
| **Phi 3 Mini** | 56.5 (54.6 - 58.5) | 100 (95 - 100) | 79.1 (59.9 - 93.7) | 100% vs 100% P>.99 | 61% vs 32% P<.001 |
| **Gemma2-27b** | 64.3 (62.4 - 66.2) | 95 (95 - 95) | 99.6 (97.4 - 99.9) | 99% vs 98% P=.002 | 95% vs 80% P<.001 |
| **Gemma2-9b** | 59.4 (57.5 - 61.4) | 90 (90 - 95) | 99.6 (96.0 - 99.9) | 92% vs 83% P<.001 | 93% vs 81% P<.001 |

**Table S4**. Effect of prompting method on the accuracy of each LLM in the US MedQA dataset (n = 2,487 questions).

| **Model** | **Expert** | **Few-shot** | **Modified Max expressed confidence** | **Non-zero Temperature** | **Vanilla** | **P value^1^** |
| --- | --- | --- | --- | --- | --- | --- |
| **GPT-3.5 Turbo** | 58.8 (56.9 - 60.8) | 63.5 (61.6 - 65.4) | 60.2 (58.3 - 62.2) | 60.3 (58.4 - 62.2) | 60.1 (58.2 - 62.0) | .01 |
| **GPT-4** | 79.5 (77.9 - 81.1) | 80.9 (79.3 - 82.4) | 80.1 (78.4 - 81.6) | 79.8 (78.1 - 81.3) | 79.3 (77.7 - 80.9) | .69 |
| **GPT-4o** | 89.4 (88.1 - 90.6) | 89.9 (88.6 - 91.0) | 88.9 (87.6 - 90.1) | 88.9 (87.6 - 90.1) | 89.0 (87.7 - 90.2) | .76 |
| **Llama3.1-70b** | 78.6 (76.9 - 80.2) | 79.0 (77.3 - 80.6) | 79.1 (77.5 - 80.7) | 79.4 (77.7 - 80.9) | 79.1 (77.4 - 80.6) | .97 |
| **Llama3.1-8b** | 62.3 (60.3 - 64.2) | 61.6 (59.7 - 63.6) | 61.7 (59.8 - 63.6) | 61.8 (59.9 - 63.8) | 62.0 (60.0 - 63.9) | >.99 |
| **Phi 3 Medium** | 67.1 (65.2 - 68.9) | 69.7 (67.9 - 71.5) | 67.8 (65.9 - 69.6) | 67.8 (65.9 - 69.6) | 67.7 (65.8 - 69.5) | .33 |
| **Phi 3 Mini** | 56.1 (54.1 - 58.0) | 56.5 (54.5 - 58.4) | 56.1 (54.1 - 58.0) | 55.9 (53.9 - 57.9) | 56.5 (54.6 - 58.5) | .99 |
| **Gemma2-27b** | 64.7 (62.8 - 66.6) | 65.9 (64.0 - 67.8) | 64.7 (62.7 - 66.5) | 64.2 (62.3 - 66.1) | 64.3 (62.4 - 66.2) | .74 |
| **Gemma2-9b** | 58.5 (56.6 - 60.5) | 60.7 (58.7 - 62.6) | 59.7 (57.7 - 61.6) | 59.1 (57.2 - 61.1) | 59.4 (57.5 - 61.4) | .64 |
| ^1^Chi-Square Test | | | | | | |

**Table S5.** Diagnostic performance accuracy of expressed confidence vs. token response probability, according to the different models (MedQA - Taiwan, n = 2,734 questions).

| **Model** | **Metric** | **Expressed confidence** | **Token probability** | ***P*** |
| --- | --- | --- | --- | --- |
| **GPT-3.5 Turbo** | AUROC *(95%CI)* | 0.52 (0.5 - 0.53) | 0.7 (0.68 - 0.72) | <.001 |
|  | Optimal threshold *(%)* | 99 | 94 |  |
|  | True positive rate *(%, 95%CI)* | 89 (87 - 90) | 55 (52 - 57) | <.001 |
|  | False positive rate *(%, 95%CI)* | 86 (84 - 87) | 23 (21 - 25) | <.001 |
|  | Correct above threshold *(%, 95%CI)* | 49 (47 - 51) | 69 (66 - 71) | <.001 |
|  | Correct below threshold *(%, 95%CI)* | 42 (37 - 47) | 36 (33 - 38) | .01 |
| **GPT-4** | AUROC *(95%CI)* | 0.57 (0.55 - 0.58) | 0.83 (0.81 - 0.85) | <.001 |
|  | Optimal threshold *(%)* | 98 | 100 |  |
|  | True positive rate *(%, 95%CI)* | 81 (79 - 82) | 68 (66 - 70) | <.001 |
|  | False positive rate *(%, 95%CI)* | 68 (65 - 71) | 14 (12 - 17) | <.001 |
|  | Correct above threshold *(%, 95%CI)* | 77 (75 - 78) | 93 (92 - 94) | <.001 |
|  | Correct below threshold *(%, 95%CI)* | 63 (59 - 67) | 51 (48 - 54) | <.001 |
| **GPT-4o** | AUROC *(95%CI)* | 0.7 (0.68 - 0.72) | 0.87 (0.85 - 0.88) | <.001 |
|  | Optimal threshold *(%)* | 92 | 100 |  |
|  | True positive rate *(%, 95%CI)* | 55 (53 - 57) | 72 (70 - 74) | <.001 |
|  | False positive rate *(%, 95%CI)* | 15 (12 - 19) | 11 (7.9 - 14) | .02 |
|  | Correct above threshold *(%, 95%CI)* | 95 (94 - 96) | 97 (97 - 98) | <.001 |
|  | Correct below threshold *(%, 95%CI)* | 74 (72 - 77) | 64 (61 - 66) | <.001 |
| **Llama3.1-8b** | AUROC *(95%CI)* | 0.47 (0.45 - 0.48) | 0.75 (0.73 - 0.77) | <.001 |
|  | Optimal threshold *(%)* | 100 | 71 |  |
|  | True positive rate *(%, 95%CI)* | 83 (81 - 85) | 63 (60 - 65) | <.001 |
|  | False positive rate *(%, 95%CI)* | 85 (83 - 86) | 22 (20 - 24) | <.001 |
|  | Correct above threshold *(%, 95%CI)* | 50 (48 - 52) | 75 (72 - 77) | <.001 |
|  | Correct below threshold *(%, 95%CI)* | 53 (48 - 57) | 33 (30 - 35) | <.001 |
| **Llama3.1-70b** | AUROC *(95%CI)* | 0.51 (0.49 - 0.53) | 0.84 (0.82 - 0.85) | <.001 |
|  | Optimal threshold *(%)* | 85 | 87 |  |
|  | True positive rate *(%, 95%CI)* | 81 (79 - 82) | 71 (69 - 73) | <.001 |
|  | False positive rate *(%, 95%CI)* | 74 (71 - 77) | 16 (13 - 18) | <.001 |
|  | Correct above threshold *(%, 95%CI)* | 69 (67 - 71) | 90 (88 - 92) | <.001 |
|  | Correct below threshold *(%, 95%CI)* | 60 (56 - 64) | 41 (39 - 44) | <.001 |
| **Phi 3 Mini** | AUROC *(95%CI)* | 0.49 (0.48 - 0.5) | 0.65 (0.63 - 0.67) | <.001 |
|  | Optimal threshold *(%)* | 100 | 71 |  |
|  | True positive rate *(%, 95%CI)* | 100 (100 - 100) | 39 (36 - 43) | <.001 |
|  | False positive rate *(%, 95%CI)* | 100 (100 - 100) | 17 (15 - 19) | <.001 |
|  | Correct above threshold *(%, 95%CI)* | 37 (35 - 39) | 58 (54 - 61) | <.001 |
|  | Correct below threshold *(%, 95%CI)* | 50 (0 - 100) | 30 (28 - 32) | .63 |
| **Phi 3 Medium** | AUROC *(95%CI)* | 0.57 (0.55 - 0.59) | 0.71 (0.69 - 0.73) | <.001 |
|  | Optimal threshold *(%)* | 92 | 87 |  |
|  | True positive rate *(%, 95%CI)* | 77 (74 - 79) | 47 (44 - 50) | <.001 |
|  | False positive rate *(%, 95%CI)* | 62 (60 - 65) | 13 (12 - 15) | <.001 |
|  | Correct above threshold *(%, 95%CI)* | 50 (48 - 52) | 74 (71 - 77) | <.001 |
|  | Correct below threshold *(%, 95%CI)* | 33 (30 - 36) | 33 (31 - 35) | .96 |
| **Gemma2-9b** | AUROC *(95%CI)* | 0.54 (0.52 - 0.56) | 0.73 (0.71 - 0.75) | <.001 |
|  | Optimal threshold *(%)* | 92 | 99 |  |
|  | True positive rate *(%, 95%CI)* | 65 (62 - 67) | 57 (54 - 59) | <.001 |
|  | False positive rate *(%, 95%CI)* | 56 (53 - 58) | 20 (18 - 22) | <.001 |
|  | Correct above threshold *(%, 95%CI)* | 55 (53 - 58) | 75 (72 - 78) | <.001 |
|  | Correct below threshold *(%, 95%CI)* | 46 (43 - 49) | 37 (34 - 39) | <.001 |
| **Gemma2-27b** | AUROC *(95%CI)* | 0.52 (0.5 - 0.54) | 0.76 (0.74 - 0.78) | <.001 |
|  | Optimal threshold *(%)* | 92 | 99 |  |
|  | True positive rate *(%, 95%CI)* | 79 (77 - 81) | 50 (48 - 53) | <.001 |
|  | False positive rate *(%, 95%CI)* | 73 (70 - 75) | 8.9 (7.3 - 10) | <.001 |
|  | Correct above threshold *(%, 95%CI)* | 56 (53 - 58) | 87 (84 - 89) | <.001 |
|  | Correct below threshold *(%, 95%CI)* | 47 (44 - 51) | 39 (37 - 41) | <.001 |

**Table S6.** Diagnostic performance accuracy of expressed confidence vs. token response probability, according to the different models (MedQA – Mainland China, n = 3,414 questions).

| **Model** | **Metric** | **Expressed confidence** | **Token probability** | ***P*** |
| --- | --- | --- | --- | --- |
| **GPT-3.5 Turbo** | AUROC *(95%CI)* | 0.51 (0.5 - 0.53) | 0.57 (0.55 - 0.59) | <.001 |
|  | Optimal threshold *(%)* | 98 | 67 |  |
|  | True positive rate *(%, 95%CI)* | 88 (86 - 90) | 61 (58 - 64) | <.001 |
|  | False positive rate *(%, 95%CI)* | 85 (84 - 86) | 51 (49 - 53) | <.001 |
|  | Correct above threshold *(%, 95%CI)* | 28 (26 - 29) | 31 (29 - 33) | <.001 |
|  | Correct below threshold *(%, 95%CI)* | 23 (20 - 27) | 23 (21 - 25) | .79 |
| **GPT-4** | AUROC *(95%CI)* | 0.52 (0.51 - 0.54) | 0.71 (0.69 - 0.73) | <.001 |
|  | Optimal threshold *(%)* | 92 | 100 |  |
|  | True positive rate *(%, 95%CI)* | 90 (88 - 91) | 63 (61 - 65) | <.001 |
|  | False positive rate *(%, 95%CI)* | 85 (83 - 87) | 30 (28 - 32) | <.001 |
|  | Correct above threshold *(%, 95%CI)* | 54 (52 - 55) | 70 (68 - 72) | <.001 |
|  | Correct below threshold *(%, 95%CI)* | 43 (38 - 47) | 37 (34 - 39) | .009 |
| **GPT-4o** | AUROC *(95%CI)* | 0.62 (0.6 - 0.63) | 0.8 (0.79 - 0.82) | <.001 |
|  | Optimal threshold *(%)* | 92 | 100 |  |
|  | True positive rate *(%, 95%CI)* | 36 (34 - 38) | 66 (64 - 68) | <.001 |
|  | False positive rate *(%, 95%CI)* | 12 (10 - 14) | 19 (16 - 21) | <.001 |
|  | Correct above threshold *(%, 95%CI)* | 87 (85 - 89) | 89 (87 - 90) | .09 |
|  | Correct below threshold *(%, 95%CI)* | 62 (60 - 64) | 48 (46 - 51) | <.001 |
| **Llama3.1-8b** | AUROC *(95%CI)* | 0.5 (0.48 - 0.52) | 0.59 (0.57 - 0.62) | <.001 |
|  | Optimal threshold *(%)* | 85 | 53 |  |
|  | True positive rate *(%, 95%CI)* | 93 (91 - 95) | 46 (43 - 49) | <.001 |
|  | False positive rate *(%, 95%CI)* | 92 (91 - 93) | 33 (31 - 35) | <.001 |
|  | Correct above threshold *(%, 95%CI)* | 27 (25 - 28) | 34 (31 - 36) | <.001 |
|  | Correct below threshold *(%, 95%CI)* | 25 (19 - 30) | 22 (21 - 24) | .41 |
| **Llama3.1-70b** | AUROC *(95%CI)* | 0.53 (0.51 - 0.54) | 0.71 (0.69 - 0.72) | <.001 |
|  | Optimal threshold *(%)* | 95 | 71 |  |
|  | True positive rate *(%, 95%CI)* | 76 (74 - 79) | 50 (47 - 53) | <.001 |
|  | False positive rate *(%, 95%CI)* | 71 (69 - 73) | 18 (17 - 20) | <.001 |
|  | Correct above threshold *(%, 95%CI)* | 43 (41 - 44) | 65 (62 - 68) | <.001 |
|  | Correct below threshold *(%, 95%CI)* | 36 (33 - 39) | 30 (28 - 31) | <.001 |
| **Phi 3 Mini** | AUROC *(95%CI)* | 0.5 (0.49 - 0.51) | 0.56 (0.54 - 0.59) | <.001 |
|  | Optimal threshold *(%)* | 92 | 44 |  |
|  | True positive rate *(%, 95%CI)* | 100 (100 - 100) | 48 (45 - 51) | <.001 |
|  | False positive rate *(%, 95%CI)* | 100 (100 - 100) | 38 (36 - 40) | <.001 |
|  | Correct above threshold *(%, 95%CI)* | 24 (23 - 26) | 29 (26 - 31) | <.001 |
|  | Correct below threshold *(%, 95%CI)* | 0 (0 - 0) | 21 (19 - 23) | <.001 |
| **Phi 3 Medium** | AUROC *(95%CI)* | 0.53 (0.51 - 0.55) | 0.58 (0.56 - 0.61) | <.001 |
|  | Optimal threshold *(%)* | 92 | 67 |  |
|  | True positive rate *(%, 95%CI)* | 63 (60 - 67) | 33 (30 - 36) | <.001 |
|  | False positive rate *(%, 95%CI)* | 58 (56 - 60) | 19 (17 - 21) | <.001 |
|  | Correct above threshold *(%, 95%CI)* | 29 (27 - 31) | 39 (36 - 42) | <.001 |
|  | Correct below threshold *(%, 95%CI)* | 24 (22 - 27) | 23 (22 - 25) | .32 |
| **Gemma2-9b** | AUROC *(95%CI)* | 0.51 (0.49 - 0.53) | 0.62 (0.6 - 0.64) | <.001 |
|  | Optimal threshold *(%)* | 98 | 88 |  |
|  | True positive rate *(%, 95%CI)* | 47 (44 - 50) | 44 (41 - 47) | .23 |
|  | False positive rate *(%, 95%CI)* | 45 (43 - 47) | 26 (24 - 27) | <.001 |
|  | Correct above threshold *(%, 95%CI)* | 30 (28 - 33) | 42 (39 - 45) | <.001 |
|  | Correct below threshold *(%, 95%CI)* | 29 (27 - 31) | 24 (22 - 26) | <.001 |
| **Gemma2-27b** | AUROC *(95%CI)* | 0.53 (0.51 - 0.55) | 0.64 (0.62 - 0.66) | <.001 |
|  | Optimal threshold *(%)* | 98 | 90 |  |
|  | True positive rate *(%, 95%CI)* | 69 (66 - 72) | 50 (47 - 53) | <.001 |
|  | False positive rate *(%, 95%CI)* | 62 (60 - 64) | 30 (28 - 32) | <.001 |
|  | Correct above threshold *(%, 95%CI)* | 34 (32 - 36) | 44 (41 - 47) | <.001 |
|  | Correct below threshold *(%, 95%CI)* | 28 (25 - 30) | 25 (23 - 26) | .01 |

**Table S7.** Diagnostic performance accuracy of expressed confidence vs. token response probability, according to the different models (MedMCQA - India, n = 2,763 questions).

| **Model** | **Metric** | **Expressed confidence** | **Token probability** | ***P*** |
| --- | --- | --- | --- | --- |
| **GPT-3.5 Turbo** | AUROC *(95%CI)* | 0.51 (0.5 - 0.51) | 0.72 (0.7 - 0.73) | <.001 |
|  | Optimal threshold *(%)* | 98 | 96 |  |
|  | True positive rate *(%, 95%CI)* | 96 (95 - 97) | 64 (61 - 66) | <.001 |
|  | False positive rate *(%, 95%CI)* | 95 (93 - 96) | 30 (28 - 33) | <.001 |
|  | Correct above threshold *(%, 95%CI)* | 57 (55 - 59) | 74 (71 - 76) | <.001 |
|  | Correct below threshold *(%, 95%CI)* | 51 (43 - 60) | 40 (38 - 43) | .02 |
| **GPT-4** | AUROC *(95%CI)* | 0.61 (0.59 - 0.63) | 0.79 (0.77 - 0.81) | <.001 |
|  | Optimal threshold *(%)* | 92 | 100 |  |
|  | True positive rate *(%, 95%CI)* | 76 (74 - 78) | 68 (66 - 70) | <.001 |
|  | False positive rate *(%, 95%CI)* | 55 (52 - 59) | 21 (18 - 24) | <.001 |
|  | Correct above threshold *(%, 95%CI)* | 78 (76 - 79) | 89 (88 - 91) | <.001 |
|  | Correct below threshold *(%, 95%CI)* | 57 (54 - 60) | 50 (48 - 53) | <.001 |
| **GPT-4o** | AUROC *(95%CI)* | 0.71 (0.69 - 0.73) | 0.83 (0.82 - 0.85) | <.001 |
|  | Optimal threshold *(%)* | 92 | 100 |  |
|  | True positive rate *(%, 95%CI)* | 47 (45 - 49) | 73 (71 - 74) | <.001 |
|  | False positive rate *(%, 95%CI)* | 8.5 (6.3 - 11) | 18 (15 - 22) | <.001 |
|  | Correct above threshold *(%, 95%CI)* | 95 (94 - 96) | 93 (92 - 94) | .010 |
|  | Correct below threshold *(%, 95%CI)* | 67 (65 - 69) | 54 (51 - 57) | <.001 |
| **Llama3.1-8b** | AUROC *(95%CI)* | 0.56 (0.54 - 0.58) | 0.72 (0.7 - 0.74) | <.001 |
|  | Optimal threshold *(%)* | 85 | 91 |  |
|  | True positive rate *(%, 95%CI)* | 87 (85 - 89) | 56 (53 - 58) | <.001 |
|  | False positive rate *(%, 95%CI)* | 75 (73 - 77) | 22 (19 - 24) | <.001 |
|  | Correct above threshold *(%, 95%CI)* | 60 (58 - 62) | 77 (75 - 80) | <.001 |
|  | Correct below threshold *(%, 95%CI)* | 41 (36 - 45) | 43 (40 - 45) | .30 |
| **Llama3.1-70b** | AUROC *(95%CI)* | 0.56 (0.54 - 0.58) | 0.82 (0.81 - 0.84) | <.001 |
|  | Optimal threshold *(%)* | 85 | 98 |  |
|  | True positive rate *(%, 95%CI)* | 88 (86 - 89) | 64 (62 - 66) | <.001 |
|  | False positive rate *(%, 95%CI)* | 72 (69 - 75) | 13 (11 - 16) | <.001 |
|  | Correct above threshold *(%, 95%CI)* | 75 (73 - 77) | 92 (91 - 94) | <.001 |
|  | Correct below threshold *(%, 95%CI)* | 52 (48 - 57) | 51 (48 - 53) | .51 |
| **Phi 3 Mini** | AUROC *(95%CI)* | 0.55 (0.53 - 0.57) | 0.71 (0.69 - 0.73) | <.001 |
|  | Optimal threshold *(%)* | 98 | 88 |  |
|  | True positive rate *(%, 95%CI)* | 56 (54 - 59) | 52 (49 - 54) | .004 |
|  | False positive rate *(%, 95%CI)* | 47 (44 - 50) | 17 (15 - 19) | <.001 |
|  | Correct above threshold *(%, 95%CI)* | 59 (56 - 61) | 78 (76 - 81) | <.001 |
|  | Correct below threshold *(%, 95%CI)* | 49 (47 - 52) | 41 (38 - 43) | <.001 |
| **Phi 3 Medium** | AUROC *(95%CI)* | 0.6 (0.58 - 0.62) | 0.75 (0.73 - 0.77) | <.001 |
|  | Optimal threshold *(%)* | 92 | 95 |  |
|  | True positive rate *(%, 95%CI)* | 82 (80 - 84) | 57 (55 - 59) | <.001 |
|  | False positive rate *(%, 95%CI)* | 64 (61 - 67) | 17 (15 - 20) | <.001 |
|  | Correct above threshold *(%, 95%CI)* | 67 (65 - 69) | 84 (82 - 86) | <.001 |
|  | Correct below threshold *(%, 95%CI)* | 44 (40 - 48) | 45 (43 - 48) | .44 |
| **Gemma2-9b** | AUROC *(95%CI)* | 0.57 (0.55 - 0.59) | 0.74 (0.72 - 0.75) | <.001 |
|  | Optimal threshold *(%)* | 92 | 100 |  |
|  | True positive rate *(%, 95%CI)* | 67 (65 - 69) | 57 (54 - 59) | <.001 |
|  | False positive rate *(%, 95%CI)* | 54 (51 - 56) | 19 (17 - 22) | <.001 |
|  | Correct above threshold *(%, 95%CI)* | 62 (60 - 64) | 79 (77 - 81) | <.001 |
|  | Correct below threshold *(%, 95%CI)* | 48 (45 - 51) | 41 (39 - 44) | <.001 |
| **Gemma2-27b** | AUROC *(95%CI)* | 0.55 (0.53 - 0.56) | 0.76 (0.74 - 0.78) | <.001 |
|  | Optimal threshold *(%)* | 98 | 99 |  |
|  | True positive rate *(%, 95%CI)* | 70 (68 - 73) | 65 (62 - 67) | <.001 |
|  | False positive rate *(%, 95%CI)* | 62 (60 - 65) | 24 (21 - 26) | <.001 |
|  | Correct above threshold *(%, 95%CI)* | 64 (61 - 66) | 81 (79 - 83) | <.001 |
|  | Correct below threshold *(%, 95%CI)* | 55 (52 - 58) | 42 (39 - 44) | <.001 |

**Table S8.** Diagnostic performance accuracy of expressed confidence vs. token response probability, according to the different models (FrMedMCQA - France, n = 1,076 questions).

| **Model** | **Metric** | **Expressed confidence** | **Token probability** | ***P*** |
| --- | --- | --- | --- | --- |
| **GPT-4** | AUROC *(95%CI)* | 0.57 (0.54 - 0.6) | 0.68 (0.64 - 0.72) | <.001 |
|  | Optimal threshold *(%)* | 98 | 90 |  |
|  | True positive rate *(%, 95%CI)* | 92 (90 - 94) | 71 (68 - 74) | <.001 |
|  | False positive rate *(%, 95%CI)* | 78 (72 - 84) | 43 (36 - 50) | <.001 |
|  | Correct above threshold *(%, 95%CI)* | 85 (83 - 88) | 89 (87 - 91) | <.001 |
|  | Correct below threshold *(%, 95%CI)* | 64 (55 - 73) | 72 (67 - 76) | .05 |
| **GPT-4o** | AUROC *(95%CI)* | 0.8 (0.76 - 0.84) | 0.77 (0.72 - 0.82) | .37 |
|  | Optimal threshold *(%)* | 92 | 100 |  |
|  | True positive rate *(%, 95%CI)* | 87 (84 - 89) | 76 (73 - 78) | <.001 |
|  | False positive rate *(%, 95%CI)* | 37 (27 - 47) | 33 (23 - 42) | .64 |
|  | Correct above threshold *(%, 95%CI)* | 96 (95 - 97) | 96 (95 - 97) | .95 |
|  | Correct below threshold *(%, 95%CI)* | 69 (62 - 75) | 79 (74 - 83) | <.001 |
| **Llama3.1-8b** | AUROC *(95%CI)* | 0.54 (0.52 - 0.57) | 0.71 (0.68 - 0.74) | <.001 |
|  | Optimal threshold *(%)* | 85 | 83 |  |
|  | True positive rate *(%, 95%CI)* | 83 (80 - 86) | 60 (56 - 64) | <.001 |
|  | False positive rate *(%, 95%CI)* | 74 (70 - 78) | 25 (21 - 29) | <.001 |
|  | Correct above threshold *(%, 95%CI)* | 57 (54 - 61) | 74 (70 - 78) | <.001 |
|  | Correct below threshold *(%, 95%CI)* | 44 (37 - 50) | 39 (35 - 43) | .13 |
| **Llama3.1-70b** | AUROC *(95%CI)* | 0.54 (0.51 - 0.56) | 0.77 (0.73 - 0.8) | <.001 |
|  | Optimal threshold *(%)* | 85 | 91 |  |
|  | True positive rate *(%, 95%CI)* | 96 (95 - 98) | 74 (71 - 77) | <.001 |
|  | False positive rate *(%, 95%CI)* | 89 (85 - 93) | 25 (19 - 30) | <.001 |
|  | Correct above threshold *(%, 95%CI)* | 78 (76 - 81) | 91 (89 - 93) | <.001 |
|  | Correct below threshold *(%, 95%CI)* | 51 (38 - 64) | 53 (48 - 58) | .73 |
| **Phi 3 Mini** | AUROC *(95%CI)* | 0.54 (0.51 - 0.57) | 0.7 (0.66 - 0.73) | <.001 |
|  | Optimal threshold *(%)* | 98 | 66 |  |
|  | True positive rate *(%, 95%CI)* | 58 (54 - 62) | 65 (61 - 69) | .01 |
|  | False positive rate *(%, 95%CI)* | 51 (46 - 55) | 33 (30 - 37) | <.001 |
|  | Correct above threshold *(%, 95%CI)* | 52 (48 - 56) | 65 (61 - 69) | <.001 |
|  | Correct below threshold *(%, 95%CI)* | 45 (40 - 49) | 33 (29 - 37) | <.001 |
| **Phi 3 Medium** | AUROC *(95%CI)* | 0.54 (0.52 - 0.57) | 0.74 (0.71 - 0.77) | <.001 |
|  | Optimal threshold *(%)* | 98 | 85 |  |
|  | True positive rate *(%, 95%CI)* | 84 (81 - 87) | 63 (59 - 66) | <.001 |
|  | False positive rate *(%, 95%CI)* | 76 (72 - 80) | 24 (19 - 28) | <.001 |
|  | Correct above threshold *(%, 95%CI)* | 66 (63 - 69) | 82 (79 - 85) | <.001 |
|  | Correct below threshold *(%, 95%CI)* | 54 (47 - 61) | 46 (42 - 50) | .03 |
| **Gemma2-9b** | AUROC *(95%CI)* | 0.58 (0.54 - 0.61) | 0.77 (0.75 - 0.8) | <.001 |
|  | Optimal threshold *(%)* | 98 | 100 |  |
|  | True positive rate *(%, 95%CI)* | 59 (56 - 63) | 66 (62 - 70) | .004 |
|  | False positive rate *(%, 95%CI)* | 46 (41 - 51) | 24 (20 - 28) | <.001 |
|  | Correct above threshold *(%, 95%CI)* | 68 (64 - 72) | 82 (78 - 85) | <.001 |
|  | Correct below threshold *(%, 95%CI)* | 55 (51 - 59) | 42 (38 - 46) | <.001 |
| **Gemma2-27b** | AUROC *(95%CI)* | 0.6 (0.57 - 0.64) | 0.8 (0.77 - 0.83) | <.001 |
|  | Optimal threshold *(%)* | 98 | 100 |  |
|  | True positive rate *(%, 95%CI)* | 62 (59 - 66) | 68 (65 - 72) | .003 |
|  | False positive rate *(%, 95%CI)* | 43 (38 - 49) | 18 (13 - 22) | <.001 |
|  | Correct above threshold *(%, 95%CI)* | 79 (76 - 82) | 91 (89 - 93) | <.001 |
|  | Correct below threshold *(%, 95%CI)* | 63 (59 - 68) | 50 (45 - 54) | <.001 |

**Table S9.** Adaptive Calibration Error and Brier score of expressed confidence vs. token response probability, according to the different models (US MedQA - n = 2,487 questions).

| **Model** | **Metric** | **Expressed confidence** | **Token probability** | **p-value** |
| --- | --- | --- | --- | --- |
| **GPT-3.5 Turbo** | Adaptive calibration error *(95%CI)* | 0.35 (0.33 - 0.38) | 0.30 (0.28 - 0.32) | <.001 |
|  | Brier score *(95%CI)* | 0.39 (0.37 - 0.41) | 0.31 (0.30 - 0.33) | <.001 |
| **GPT-4** | Adaptive calibration error *(95%CI)* | 0.14 (0.11 - 0.16) | 0.18 (0.16 - 0.19) | <.001 |
|  | Brier score *(95%CI)* | 0.19 (0.18 - 0.20) | 0.18 (0.17 - 0.19) | <.001 |
| **GPT-4o** | Adaptive calibration error *(95%CI)* | 0.05 (0.03 - 0.06) | 0.08 (0.07 - 0.09) | <.001 |
|  | Brier score *(95%CI)* | 0.10 (0.09 - 0.11) | 0.09 (0.08 - 0.11) | .17 |
| **Llama3.1-8b** | Adaptive calibration error *(95%CI)* | 0.25 (0.23 - 0.27) | 0.21 (0.19 - 0.23) | <.001 |
|  | Brier score *(95%CI)* | 0.31 (0.29 - 0.32) | 0.25 (0.24 - 0.26) | <.001 |
| **Llama3.1-70b** | Adaptive calibration error *(95%CI)* | 0.09 (0.07 - 0.11) | 0.12 (0.11 - 0.13) | <.001 |
|  | Brier score *(95%CI)* | 0.18 (0.17 - 0.20) | 0.15 (0.14 - 0.16) | <.001 |
| **Phi 3 Mini** | Adaptive calibration error *(95%CI)* | 0.39 (0.37 - 0.41) | 0.19 (0.18 - 0.22) | <.001 |
|  | Brier score *(95%CI)* | 0.41 (0.40 - 0.43) | 0.25 (0.24 - 0.27) | <.001 |
| **Phi 3 Medium** | Adaptive calibration error *(95%CI)* | 0.23 (0.22 - 0.26) | 0.17 (0.15 - 0.19) | <.001 |
|  | Brier score *(95%CI)* | 0.29 (0.27 - 0.30) | 0.21 (0.20 - 0.22) | <.001 |
| **Gemma2-9b** | Adaptive calibration error *(95%CI)* | 0.29 (0.27 - 0.31) | 0.34 (0.33 - 0.37) | <.001 |
|  | Brier score *(95%CI)* | 0.33 (0.32 - 0.35) | 0.35 (0.33 - 0.36) | <.001 |
| **Gemma2-27b** | Adaptive calibration error *(95%CI)* | 0.27 (0.25 - 0.29) | 0.30 (0.29 - 0.32) | <.001 |
|  | Brier score *(95%CI)* | 0.32 (0.30 - 0.33) | 0.30 (0.29 - 0.32) | <.001 |

**Table S10.** Adaptive Calibration Error and Brier score of expressed confidence vs. token response probability, according to the different models (Taiwan MedQA - n = 2,734 questions).

| **Model** | **Metric** | **Expressed confidence** | **Token probability** | **p-value** |
| --- | --- | --- | --- | --- |
| **GPT-3.5 Turbo** | Adaptive calibration error (95%CI) | 0.45 (0.42 - 0.49) | 0.31 (0.29 - 0.33) | <.001 |
|  | Brier score (95%CI) | 0.50 (0.49 - 0.52) | 0.33 (0.31 - 0.34) | <.001 |
| **GPT-4** | Adaptive calibration error (95%CI) | 0.21 (0.19 - 0.23) | 0.21 (0.20 - 0.23) | .70 |
|  | Brier score (95%CI) | 0.25 (0.23 - 0.27) | 0.22 (0.20 - 0.23) | <.001 |
| **GPT-4o** | Adaptive calibration error (95%CI) | 0.15 (0.03 - 0.18) | 0.12 (0.10 - 0.13) | .27 |
|  | Brier score (95%CI) | 0.13 (0.12 - 0.14) | 0.13 (0.11 - 0.14) | .004 |
| **Llama3.1-8b** | Adaptive calibration error (95%CI) | 0.42 (0.40 - 0.44) | 0.16 (0.15 - 0.18) | <.001 |
|  | Brier score (95%CI) | 0.47 (0.45 - 0.48) | 0.23 (0.22 - 0.24) | <.001 |
| **Llama3.1-70b** | Adaptive calibration error (95%CI) | 0.25 (0.23 - 0.27) | 0.12 (0.11 - 0.14) | <.001 |
|  | Brier score (95%CI) | 0.31 (0.29 - 0.32) | 0.17 (0.17 - 0.18) | <.001 |
| **Phi 3 Mini** | Adaptive calibration error (95%CI) | 0.56 (0.53 - 0.61) | 0.20 (0.19 - 0.22) | <.001 |
|  | Brier score (95%CI) | 0.62 (0.61 - 0.64) | 0.26 (0.25 - 0.27) | <.001 |
| **Phi 3 Medium** | Adaptive calibration error (95%CI) | 0.47 (0.45 - 0.49) | 0.23 (0.21 - 0.25) | <.001 |
|  | Brier score (95%CI) | 0.50 (0.48 - 0.51) | 0.27 (0.26 - 0.28) | <.001 |
| **Gemma2-9b** | Adaptive calibration error (95%CI) | 0.39 (0.36 - 0.42) | 0.33 (0.32 - 0.35) | <.001 |
|  | Brier score (95%CI) | 0.43 (0.42 - 0.45) | 0.34 (0.33 - 0.36) | <.001 |
| **Gemma2-27b** | Adaptive calibration error (95%CI) | 0.35 (0.30 - 0.42) | 0.35 (0.33 - 0.37) | .45 |
|  | Brier score (95%CI) | 0.43 (0.42 - 0.45) | 0.35 (0.33 - 0.36) | <.001 |

**Table S11.** Adaptive Calibration Error and Brier score of expressed confidence vs. token response probability, according to the different models (Mainland China MedQA, n = 3,414 questions).

| **Model** | **Metric** | **Expressed confidence** | **Token probability** | **p-value** |
| --- | --- | --- | --- | --- |
| **GPT-3.5 Turbo** | Adaptive calibration error (95%CI) | 0.70 (0.68 - 0.71) | 0.42 (0.41 - 0.44) | <.001 |
|  | Brier score (95%CI) | 0.71 (0.69 - 0.72) | 0.40 (0.39 - 0.41) | <.001 |
| **GPT-4** | Adaptive calibration error (95%CI) | 0.42 (0.40 - 0.45) | 0.39 (0.37 - 0.41) | <.001 |
|  | Brier score (95%CI) | 0.46 (0.45 - 0.48) | 0.39 (0.38 - 0.41) | <.001 |
| **GPT-4o** | Adaptive calibration error (95%CI) | 0.20 (0.18 - 0.24) | 0.23 (0.21 - 0.24) | .007 |
|  | Brier score (95%CI) | 0.26 (0.25 - 0.27) | 0.23 (0.22 - 0.25) | <.001 |
| **Llama3.1-8b** | Adaptive calibration error (95%CI) | 0.66 (0.64 - 0.68) | 0.24 (0.23 - 0.25) | <.001 |
|  | Brier score (95%CI) | 0.69 (0.68 - 0.71) | 0.26 (0.25 - 0.27) | <.001 |
| **Llama3.1-70b** | Adaptive calibration error (95%CI) | 0.46 (0.41 - 0.53) | 0.19 (0.18 - 0.21) | <.001 |
|  | Brier score (95%CI) | 0.54 (0.53 - 0.56) | 0.25 (0.24 - 0.25) | <.001 |
| **Phi 3 Mini** | Adaptive calibration error (95%CI) | 0.73 (0.72 - 0.75) | 0.20 (0.18 - 0.21) | <.001 |
|  | Brier score (95%CI) | 0.75 (0.74 - 0.77) | 0.23 (0.22 - 0.23) | <.001 |
| **Phi 3 Medium** | Adaptive calibration error (95%CI) | 0.65 (0.63 - 0.67) | 0.27 (0.25 - 0.28) | <.001 |
|  | Brier score (95%CI) | 0.64 (0.63 - 0.65) | 0.28 (0.27 - 0.28) | <.001 |
| **Gemma2-9b** | Adaptive calibration error (95%CI) | 0.62 (0.60 - 0.63) | 0.40 (0.38 - 0.41) | <.001 |
|  | Brier score (95%CI) | 0.63 (0.62 - 0.65) | 0.38 (0.37 - 0.39) | <.001 |
| **Gemma2-27b** | Adaptive calibration error (95%CI) | 0.63 (0.61 - 0.64) | 0.45 (0.44 - 0.47) | <.001 |
|  | Brier score (95%CI) | 0.65 (0.63 - 0.66) | 0.43 (0.42 - 0.44) | <.001 |

**Table S12.** Adaptive Calibration Error and Brier score of expressed confidence vs. token response probability, according to the different models (India MedMCQA - n = 2,763 questions).

| **Model** | **Metric** | **Expressed confidence** | **Token probability** | **p-value** |
| --- | --- | --- | --- | --- |
| **GPT-3.5 Turbo** | Adaptive calibration error (95%CI) | 0.38 (0.36 - 0.41) | 0.28 (0.27 - 0.30) | <.001 |
|  | Brier score (95%CI) | 0.43 (0.41 - 0.45) | 0.30 (0.29 - 0.32) | <.001 |
| **GPT-4** | Adaptive calibration error (95%CI) | 0.22 (0.20 - 0.24) | 0.24 (0.22 - 0.25) | <.001 |
|  | Brier score (95%CI) | 0.26 (0.24 - 0.28) | 0.24 (0.23 - 0.26) | <.001 |
| **GPT-4o** | Adaptive calibration error (95%CI) | 0.13 (0.11 - 0.14) | 0.16 (0.15 - 0.17) | <.001 |
|  | Brier score (95%CI) | 0.18 (0.17 - 0.20) | 0.17 (0.16 - 0.18) | <.001 |
| **Llama3.1-8b** | Adaptive calibration error (95%CI) | 0.33 (0.31 - 0.35) | 0.20 (0.18 - 0.22) | <.001 |
|  | Brier score (95%CI) | 0.37 (0.36 - 0.39) | 0.26 (0.25 - 0.27) | <.001 |
| **Llama3.1-70b** | Adaptive calibration error (95%CI) | 0.18 (0.16 - 0.20) | 0.15 (0.14 - 0.17) | .008 |
|  | Brier score (95%CI) | 0.26 (0.25 - 0.28) | 0.19 (0.18 - 0.20) | <.001 |
| **Phi 3 Mini** | Adaptive calibration error (95%CI) | 0.41 (0.39 - 0.43) | 0.20 (0.19 - 0.22) | <.001 |
|  | Brier score (95%CI) | 0.43 (0.42 - 0.45) | 0.26 (0.25 - 0.27) | <.001 |
| **Phi 3 Medium** | Adaptive calibration error (95%CI) | 0.25 (0.19 - 0.32) | 0.20 (0.19 - 0.22) | .96 |
|  | Brier score (95%CI) | 0.34 (0.33 - 0.36) | 0.24 (0.23 - 0.25) | <.001 |
| **Gemma2-9b** | Adaptive calibration error (95%CI) | 0.33 (0.31 - 0.36) | 0.35 (0.34 - 0.37) | .02 |
|  | Brier score (95%CI) | 0.39 (0.37 - 0.40) | 0.36 (0.34 - 0.37) | <.001 |
| **Gemma2-27b** | Adaptive calibration error (95%CI) | 0.34 (0.32 - 0.35) | 0.32 (0.31 - 0.34) | <.001 |
|  | Brier score (95%CI) | 0.37 (0.35 - 0.39) | 0.33 (0.31 - 0.34) | <.001 |

**Table S13.** Adaptive Calibration Error and Brier score of expressed confidence vs. token response probability, according to the different models (French MedMCQA - n = 1,076 questions).

| **Model** | **Metric** | **Expressed confidence** | **Token probability** | **p-value** |
| --- | --- | --- | --- | --- |
| **GPT-4** | Adaptive calibration error (95%CI) | 0.12 (0.09 - 0.15) | 0.11 (0.09 - 0.12) | .009 |
|  | Brier score (95%CI) | 0.16 (0.14 - 0.18) | 0.14 (0.12 - 0.16) | <.001 |
| **GPT-4o** | Adaptive calibration error (95%CI) | 0.04 (0.03 - 0.06) | 0.07 (0.05 - 0.08) | .04 |
|  | Brier score (95%CI) | 0.08 (0.06 - 0.09) | 0.08 (0.06 - 0.09) | .91 |
| **Llama3.1-8b** | Adaptive calibration error (95%CI) | 0.38 (0.35 - 0.41) | 0.20 (0.18 - 0.23) | <.001 |
|  | Brier score (95%CI) | 0.41 (0.38 - 0.44) | 0.26 (0.24 - 0.27) | <.001 |
| **Llama3.1-70b** | Adaptive calibration error (95%CI) | 0.14 (0.09 - 0.19) | 0.12 (0.10 - 0.14) | .88 |
|  | Brier score (95%CI) | 0.22 (0.20 - 0.25) | 0.15 (0.13 - 0.17) | <.001 |
| **Phi 3 Mini** | Adaptive calibration error (95%CI) | 0.48 (0.44 - 0.51) | 0.20 (0.17 - 0.22) | <.001 |
|  | Brier score (95%CI) | 0.49 (0.46 - 0.52) | 0.25 (0.24 - 0.27) | <.001 |
| **Phi 3 Medium** | Adaptive calibration error (95%CI) | 0.33 (0.30 - 0.36) | 0.15 (0.13 - 0.18) | <.001 |
|  | Brier score (95%CI) | 0.35 (0.33 - 0.38) | 0.22 (0.20 - 0.23) | <.001 |
| **Gemma2-9b** | Adaptive calibration error (95%CI) | 0.32 (0.29 - 0.35) | 0.31 (0.28 - 0.34) | .32 |
|  | Brier score (95%CI) | 0.35 (0.33 - 0.38) | 0.31 (0.29 - 0.34) | <.001 |
| **Gemma2-27b** | Adaptive calibration error (95%CI) | 0.22 (0.19 - 0.25) | 0.23 (0.21 - 0.26) | .005 |
|  | Brier score (95%CI) | 0.26 (0.23 - 0.29) | 0.24 (0.22 - 0.27) | <.001 |

**Table S14.** Accuracy of each model by knowledge type (USMedQA dataset, n = 2,487).

| **Model** | **Basic sciences and mechanisms *N=1344 (%, 95%CI)*** | **Clinical knowledge *N=1142 (%, 95%CI)*** | ***P*** |
| --- | --- | --- | --- |
| **Gemma2-27b** | 65.0 (62.4 - 67.6) | 63.5 (60.6 - 66.3) | .45 |
| **Gemma2-9b** | 59.1 (56.4 - 61.7) | 59.8 (56.9 - 62.7) | .74 |
| **GPT-3.5 Turbo** | 59.3 (56.6 - 61.9) | 61.0 (58.1 - 63.9) | .40 |
| **GPT-4** | 78.8 (76.5 - 81.0) | 79.9 (77.5 - 82.2) | .51 |
| **GPT-4o** | 88.9 (87.1 - 90.5) | 89.1 (87.1 - 90.8) | .96 |
| **Llama3.1-70b** | 78.3 (76.0 - 80.5) | 79.9 (77.4 - 82.2) | .38 |
| **Llama3.1-8b** | 60.9 (58.2 - 63.5) | 63.2 (60.4 - 66.0) | .24 |
| **Phi 3 Medium** | 68.8 (66.3 - 71.3) | 66.4 (63.6 - 69.1) | .21 |
| **Phi 3 Mini** | 55.3 (52.6 - 58.0) | 58.0 (55.0 - 60.9) | .19 |

**Table S15.** Diagnostic performance accuracy of expressed confidence vs. token response probability, by knowledge type (USMedQA dataset, n = 2,487).

| **Model** | **Uncertainty metric** | **Basic sciences and mechanisms *N=1344 (AUROC, 95%CI)*** | **Clinical knowledge *N=1142 (AUROC, 95%CI)*** | ***P*** |
| --- | --- | --- | --- | --- |
| **GPT-3.5 Turbo** | Expressed confidence | 0.51 (0.53 - 0.54) | 0.50 (0.52 - 0.53) | .37 |
|  | Token probability | 0.67 (0.70 - 0.73) | 0.66 (0.69 - 0.72) | .78 |
| **GPT-4** | Expressed confidence | 0.57 (0.61 - 0.64) | 0.57 (0.61 - 0.65) | .91 |
|  | Token probability | 0.80 (0.82 - 0.85) | 0.79 (0.82 - 0.85) | .92 |
| **GPT-4o** | Expressed confidence | 0.65 (0.69 - 0.74) | 0.66 (0.71 - 0.75) | .67 |
|  | Token probability | 0.84 (0.87 - 0.90) | 0.85 (0.88 - 0.91) | .68 |
| **Llama3.1-8b** | Expressed confidence | 0.52 (0.55 - 0.58) | 0.54 (0.57 - 0.60) | .45 |
|  | Token probability | 0.70 (0.72 - 0.75) | 0.70 (0.73 - 0.76) | .67 |
| **Llama3.1-70b** | Expressed confidence | 0.51 (0.55 - 0.59) | 0.57 (0.61 - 0.65) | .045 |
|  | Token probability | 0.81 (0.84 - 0.86) | 0.81 (0.84 - 0.86) | .99 |
| **Phi 3 Mini** | Expressed confidence | 0.48 (0.51 - 0.54) | 0.49 (0.52 - 0.54) | .75 |
|  | Token probability | 0.70 (0.73 - 0.76) | 0.66 (0.69 - 0.72) | .07 |
| **Phi 3 Medium** | Expressed confidence | 0.53 (0.55 - 0.58) | 0.55 (0.59 - 0.62) | .14 |
|  | Token probability | 0.72 (0.75 - 0.78) | 0.74 (0.77 - 0.80) | .31 |
| **Gemma2-9b** | Expressed confidence | 0.56 (0.59 - 0.62) | 0.57 (0.60 - 0.63) | .48 |
|  | Token probability | 0.71 (0.74 - 0.77) | 0.70 (0.73 - 0.76) | .54 |
| **Gemma2-27b** | Expressed confidence | 0.53 (0.56 - 0.58) | 0.53 (0.56 - 0.59) | .90 |
|  | Token probability | 0.74 (0.76 - 0.79) | 0.74 (0.77 - 0.79) | .78 |

**Table S16.** Adaptive calibration error of expressed confidence vs. token response probability, by knowledge type (USMedQA dataset, n = 2,487).

| **Model** | **Uncertainty metric** | **Basic sciences and mechanisms *N=1344 (ACE, 95%CI)*** | **Clinical knowledge *N=1142 (ACE, 95%CI)*** | ***P*** |
| --- | --- | --- | --- | --- |
| **GPT-3.5 Turbo** | Expressed confidence | 0.33 (0.32 - 0.35) | 0.33 (0.31 - 0.35) | .64 |
|  | Token probability | 0.29 (0.28 - 0.30) | 0.29 (0.28 - 0.30) | .94 |
| **GPT-4** | Expressed confidence | 0.14 (0.12 - 0.16) | 0.12 (0.11 - 0.13) | .06 |
|  | Token probability | 0.18 (0.17 - 0.19) | 0.17 (0.15 - 0.18) | .03 |
| **GPT-4o** | Expressed confidence | 0.05 (0.03 - 0.06) | 0.06 (0.05 - 0.07) | .03 |
|  | Token probability | 0.08 (0.08 - 0.09) | 0.08 (0.07 - 0.09) | .22 |
| **Gemma2-27b** | Expressed confidence | 0.26 (0.24 - 0.27) | 0.27 (0.25 - 0.28) | .69 |
|  | Token probability | 0.29 (0.28 - 0.30) | 0.31 (0.30 - 0.32) | .02 |
| **Gemma2-9b** | Expressed confidence | 0.31 (0.30 - 0.32) | 0.31 (0.29 - 0.32) | .76 |
|  | Token probability | 0.35 (0.33 - 0.36) | 0.35 (0.33 - 0.36) | .80 |
| **Llama3.1-70b** | Expressed confidence | 0.11 (0.10 - 0.12) | 0.09 (0.08 - 0.10) | .03 |
|  | Token probability | 0.14 (0.13 - 0.14) | 0.14 (0.13 - 0.15) | .80 |
| **Llama3.1-8b** | Expressed confidence | 0.25 (0.23 - 0.28) | 0.23 (0.20 - 0.26) | .30 |
|  | Token probability | 0.24 (0.23 - 0.25) | 0.23 (0.21 - 0.24) | .08 |
| **Phi 3 Medium** | Expressed confidence | 0.26 (0.25 - 0.27) | 0.27 (0.25 - 0.28) | .27 |
|  | Token probability | 0.15 (0.14 - 0.17) | 0.17 (0.16 - 0.19) | .02 |
| **Phi 3 Mini** | Expressed confidence | 0.39 (0.38 - 0.41) | 0.38 (0.37 - 0.40) | .29 |
|  | Token probability | 0.20 (0.19 - 0.22) | 0.19 (0.18 - 0.20) | .25 |

**Table S17.** Effect of prompting method on the predictive performance of the token response probability (USMedQA dataset, n = 2,487)

| **Model** | **Options** | **AUC** | ***P*** |
| --- | --- | --- | --- |
| **GPT-3.5 Turbo** | Expert vs Fewshot | 0.70 (0.68 - 0.72) vs 0.74 (0.72 - 0.76) | .005 |
|  | Expert vs Modified Max Expressed Confidence | 0.70 (0.68 - 0.72) vs 0.69 (0.67 - 0.71) | .65 |
|  | Expert vs Non-zero Temperature | 0.70 (0.68 - 0.72) vs 0.70 (0.67 - 0.72) | .72 |
|  | Expert vs Vanilla | 0.70 (0.68 - 0.72) vs 0.70 (0.68 - 0.72) | .82 |
|  | Fewshot vs Modified Max Expressed Confidence | 0.74 (0.72 - 0.76) vs 0.69 (0.67 - 0.71) | .001 |
|  | Fewshot vs Non-zero Temperature | 0.74 (0.72 - 0.76) vs 0.70 (0.67 - 0.72) | .002 |
|  | Fewshot vs Vanilla | 0.74 (0.72 - 0.76) vs 0.70 (0.68 - 0.72) | .002 |
|  | Modified Max Expressed Confidence vs Non-zero Temperature | 0.69 (0.67 - 0.71) vs 0.70 (0.67 - 0.72) | .92 |
|  | Modified Max Expressed Confidence vs Vanilla | 0.69 (0.67 - 0.71) vs 0.70 (0.68 - 0.72) | .82 |
|  | Non-zero Temperature vs Vanilla | 0.70 (0.67 - 0.72) vs 0.70 (0.68 - 0.72) | .90 |
| **GPT-4** | Expert vs Fewshot | 0.81 (0.79 - 0.83) vs 0.83 (0.81 - 0.85) | .08 |
|  | Expert vs Modified Max Expressed Confidence | 0.81 (0.79 - 0.83) vs 0.81 (0.79 - 0.83) | .66 |
|  | Expert vs Non-zero Temperature | 0.81 (0.79 - 0.83) vs 0.82 (0.80 - 0.84) | .52 |
|  | Expert vs Vanilla | 0.81 (0.79 - 0.83) vs 0.82 (0.81 - 0.84) | .25 |
|  | Fewshot vs Modified Max Expressed Confidence | 0.83 (0.81 - 0.85) vs 0.81 (0.79 - 0.83) | .19 |
|  | Fewshot vs Non-zero Temperature | 0.83 (0.81 - 0.85) vs 0.82 (0.80 - 0.84) | .28 |
|  | Fewshot vs Vanilla | 0.83 (0.81 - 0.85) vs 0.82 (0.81 - 0.84) | .57 |
|  | Modified Max Expressed Confidence vs Non-zero Temperature | 0.81 (0.79 - 0.83) vs 0.82 (0.80 - 0.84) | .83 |
|  | Modified Max Expressed Confidence vs Vanilla | 0.81 (0.79 - 0.83) vs 0.82 (0.81 - 0.84) | .47 |
|  | Non-zero Temperature vs Vanilla | 0.82 (0.80 - 0.84) vs 0.82 (0.81 - 0.84) | .61 |
| **GPT-4o** | Expert vs Fewshot | 0.87 (0.85 - 0.89) vs 0.89 (0.87 - 0.91) | .21 |
|  | Expert vs Modified Max Expressed Confidence | 0.87 (0.85 - 0.89) vs 0.88 (0.85 - 0.90) | .88 |
|  | Expert vs Non-zero Temperature | 0.87 (0.85 - 0.89) vs 0.87 (0.85 - 0.89) | .87 |
|  | Expert vs Vanilla | 0.87 (0.85 - 0.89) vs 0.87 (0.85 - 0.89) | .95 |
|  | Fewshot vs Modified Max Expressed Confidence | 0.89 (0.87 - 0.91) vs 0.88 (0.85 - 0.90) | .28 |
|  | Fewshot vs Non-zero Temperature | 0.89 (0.87 - 0.91) vs 0.87 (0.85 - 0.89) | .15 |
|  | Fewshot vs Vanilla | 0.89 (0.87 - 0.91) vs 0.87 (0.85 - 0.89) | .23 |
|  | Modified Max Expressed Confidence vs Non-zero Temperature | 0.88 (0.85 - 0.90) vs 0.87 (0.85 - 0.89) | .75 |
|  | Modified Max Expressed Confidence vs Vanilla | 0.88 (0.85 - 0.90) vs 0.87 (0.85 - 0.89) | .93 |
|  | Non-zero Temperature vs Vanilla | 0.87 (0.85 - 0.89) vs 0.87 (0.85 - 0.89) | .81 |
| **Llama3.1-8b** | Expert vs Fewshot | 0.73 (0.71 - 0.75) vs 0.76 (0.74 - 0.78) | .01 |
|  | Expert vs Modified Max Expressed Confidence | 0.73 (0.71 - 0.75) vs 0.74 (0.72 - 0.75) | .66 |
|  | Expert vs Vanilla | 0.73 (0.71 - 0.75) vs 0.73 (0.71 - 0.75) | .91 |
|  | Fewshot vs Modified Max Expressed Confidence | 0.76 (0.74 - 0.78) vs 0.74 (0.72 - 0.75) | .04 |
|  | Fewshot vs Vanilla | 0.76 (0.74 - 0.78) vs 0.73 (0.71 - 0.75) | .01 |
|  | Modified Max Expressed Confidence vs Vanilla | 0.74 (0.72 - 0.75) vs 0.73 (0.71 - 0.75) | .57 |
| **Llama3.1-70b** | Expert vs Fewshot | 0.84 (0.83 - 0.86) vs 0.86 (0.84 - 0.87) | .34 |
|  | Expert vs Modified Max Expressed Confidence | 0.84 (0.83 - 0.86) vs 0.83 (0.82 - 0.85) | .36 |
|  | Expert vs Vanilla | 0.84 (0.83 - 0.86) vs 0.84 (0.82 - 0.85) | .55 |
|  | Fewshot vs Modified Max Expressed Confidence | 0.86 (0.84 - 0.87) vs 0.83 (0.82 - 0.85) | .06 |
|  | Fewshot vs Vanilla | 0.86 (0.84 - 0.87) vs 0.84 (0.82 - 0.85) | .12 |
|  | Modified Max Expressed Confidence vs Vanilla | 0.83 (0.82 - 0.85) vs 0.84 (0.82 - 0.85) | .76 |
| **Phi 3 Mini** | Expert vs Fewshot | 0.72 (0.70 - 0.74) vs 0.72 (0.70 - 0.74) | .69 |
|  | Expert vs Modified Max Expressed Confidence | 0.72 (0.70 - 0.74) vs 0.72 (0.70 - 0.74) | .89 |
|  | Expert vs Vanilla | 0.72 (0.70 - 0.74) vs 0.71 (0.69 - 0.73) | .77 |
|  | Fewshot vs Modified Max Expressed Confidence | 0.72 (0.70 - 0.74) vs 0.72 (0.70 - 0.74) | .80 |
|  | Fewshot vs Vanilla | 0.72 (0.70 - 0.74) vs 0.71 (0.69 - 0.73) | .49 |
|  | Modified Max Expressed Confidence vs Vanilla | 0.72 (0.70 - 0.74) vs 0.71 (0.69 - 0.73) | .67 |
| **Phi 3 Medium** | Expert vs Fewshot | 0.75 (0.73 - 0.77) vs 0.76 (0.74 - 0.78) | .66 |
|  | Expert vs Modified Max Expressed Confidence | 0.75 (0.73 - 0.77) vs 0.75 (0.73 - 0.77) | .94 |
|  | Expert vs Vanilla | 0.75 (0.73 - 0.77) vs 0.76 (0.74 - 0.78) | .64 |
|  | Fewshot vs Modified Max Expressed Confidence | 0.76 (0.74 - 0.78) vs 0.75 (0.73 - 0.77) | .61 |
|  | Fewshot vs Vanilla | 0.76 (0.74 - 0.78) vs 0.76 (0.74 - 0.78) | .98 |
|  | Modified Max Expressed Confidence vs Vanilla | 0.75 (0.73 - 0.77) vs 0.76 (0.74 - 0.78) | .59 |
| **Gemma2-9b** | Expert vs Fewshot | 0.74 (0.72 - 0.76) vs 0.74 (0.72 - 0.76) | .87 |
|  | Expert vs Modified Max Expressed Confidence | 0.74 (0.72 - 0.76) vs 0.73 (0.71 - 0.75) | .57 |
|  | Expert vs Vanilla | 0.74 (0.72 - 0.76) vs 0.74 (0.72 - 0.75) | .74 |
|  | Fewshot vs Modified Max Expressed Confidence | 0.74 (0.72 - 0.76) vs 0.73 (0.71 - 0.75) | .47 |
|  | Fewshot vs Vanilla | 0.74 (0.72 - 0.76) vs 0.74 (0.72 - 0.75) | .63 |
|  | Modified Max Expressed Confidence vs Vanilla | 0.73 (0.71 - 0.75) vs 0.74 (0.72 - 0.75) | .81 |
| **Gemma2-27b** | Expert vs Fewshot | 0.74 (0.73 - 0.76) vs 0.75 (0.73 - 0.77) | .73 |
|  | Expert vs Modified Max Expressed Confidence | 0.74 (0.73 - 0.76) vs 0.76 (0.74 - 0.78) | .40 |
|  | Expert vs Vanilla | 0.74 (0.73 - 0.76) vs 0.76 (0.74 - 0.78) | .17 |
|  | Fewshot vs Modified Max Expressed Confidence | 0.75 (0.73 - 0.77) vs 0.76 (0.74 - 0.78) | .63 |
|  | Fewshot vs Vanilla | 0.75 (0.73 - 0.77) vs 0.76 (0.74 - 0.78) | .31 |
|  | Modified Max Expressed Confidence vs Vanilla | 0.76 (0.74 - 0.78) vs 0.76 (0.74 - 0.78) | .59 |

**Table S18.** Diagnostic performance accuracy comparison between uncertainty metrics, by model (USMedQA dataset, n = 2,487)

| **Model** | **Uncertainty metrics** | **AUC** | ***P*** |
| --- | --- | --- | --- |
| **GPT-3.5 Turbo** | Expressed confidence vs Perplexity | 0.52 (0.51 - 0.53) vs 0.58 (0.56 - 0.61) | <.001 |
|  | Perplexity vs Token probability | 0.58 (0.56 - 0.61) vs 0.70 (0.68 - 0.72) | <.001 |
|  | Entropy vs Expressed confidence | 0.70 (0.68 - 0.72) vs 0.52 (0.51 - 0.53) | <.001 |
|  | Entropy vs Perplexity | 0.70 (0.68 - 0.72) vs 0.58 (0.56 - 0.61) | <.001 |
|  | Entropy vs Token probability | 0.70 (0.68 - 0.72) vs 0.70 (0.68 - 0.72) | <.001 |
| **GPT-4** | Expressed confidence vs Perplexity | 0.61 (0.58 - 0.63) vs 0.73 (0.71 - 0.76) | <.001 |
|  | Perplexity vs Token probability | 0.73 (0.71 - 0.76) vs 0.82 (0.81 - 0.84) | <.001 |
|  | Entropy vs Expressed confidence | 0.82 (0.81 - 0.84) vs 0.61 (0.58 - 0.63) | <.001 |
|  | Entropy vs Perplexity | 0.82 (0.81 - 0.84) vs 0.73 (0.71 - 0.76) | <.001 |
|  | Entropy vs Token probability | 0.82 (0.81 - 0.84) vs 0.82 (0.81 - 0.84) | .41 |
| **GPT-4o** | Expressed confidence vs Perplexity | 0.70 (0.67 - 0.73) vs 0.67 (0.64 - 0.71) | .21 |
|  | Perplexity vs Token probability | 0.67 (0.64 - 0.71) vs 0.87 (0.85 - 0.89) | <.001 |
|  | Entropy vs Expressed confidence | 0.87 (0.85 - 0.89) vs 0.70 (0.67 - 0.73) | <.001 |
|  | Entropy vs Perplexity | 0.87 (0.85 - 0.89) vs 0.67 (0.64 - 0.71) | <.001 |
|  | Entropy vs Token probability | 0.87 (0.85 - 0.89) vs 0.87 (0.85 - 0.89) | .16 |
| **Llama3.1-8b** | Expressed confidence vs Perplexity | 0.56 (0.54 - 0.58) vs 0.64 (0.61 - 0.66) | <.001 |
|  | Perplexity vs Token probability | 0.64 (0.61 - 0.66) vs 0.73 (0.71 - 0.75) | <.001 |
|  | Entropy vs Expressed confidence | 0.73 (0.71 - 0.75) vs 0.56 (0.54 - 0.58) | <.001 |
|  | Entropy vs Perplexity | 0.73 (0.71 - 0.75) vs 0.64 (0.61 - 0.66) | <.001 |
|  | Entropy vs Token probability | 0.73 (0.71 - 0.75) vs 0.73 (0.71 - 0.75) | .50 |
| **Llama3.1-70b** | Expressed confidence vs Perplexity | 0.58 (0.55 - 0.60) vs 0.77 (0.75 - 0.79) | <.001 |
|  | Perplexity vs Token probability | 0.77 (0.75 - 0.79) vs 0.84 (0.82 - 0.85) | <.001 |
|  | Entropy vs Expressed confidence | 0.84 (0.83 - 0.86) vs 0.58 (0.55 - 0.60) | <.001 |
|  | Entropy vs Perplexity | 0.84 (0.83 - 0.86) vs 0.77 (0.75 - 0.79) | <.001 |
|  | Entropy vs Token probability | 0.84 (0.83 - 0.86) vs 0.84 (0.82 - 0.85) | <.001 |
| **Phi 3 Mini** | Expressed confidence vs Perplexity | 0.51 (0.49 - 0.53) vs 0.60 (0.58 - 0.62) | <.001 |
|  | Perplexity vs Token probability | 0.60 (0.58 - 0.62) vs 0.71 (0.69 - 0.73) | <.001 |
|  | Entropy vs Expressed confidence | 0.72 (0.70 - 0.74) vs 0.51 (0.49 - 0.53) | <.001 |
|  | Entropy vs Perplexity | 0.72 (0.70 - 0.74) vs 0.60 (0.58 - 0.62) | <.001 |
|  | Entropy vs Token probability | 0.72 (0.70 - 0.74) vs 0.71 (0.69 - 0.73) | .002 |
| **Phi 3 Medium** | Expressed confidence vs Perplexity | 0.57 (0.55 - 0.59) vs 0.71 (0.69 - 0.73) | <.001 |
|  | Perplexity vs Token probability | 0.71 (0.69 - 0.73) vs 0.76 (0.74 - 0.78) | <.001 |
|  | Entropy vs Expressed confidence | 0.76 (0.74 - 0.78) vs 0.57 (0.55 - 0.59) | <.001 |
|  | Entropy vs Perplexity | 0.76 (0.74 - 0.78) vs 0.71 (0.69 - 0.73) | <.001 |
|  | Entropy vs Token probability | 0.76 (0.74 - 0.78) vs 0.76 (0.74 - 0.78) | .58 |
| **Gemma2-9b** | Expressed confidence vs Perplexity | 0.60 (0.57 - 0.62) vs 0.62 (0.60 - 0.64) | .02 |
|  | Perplexity vs Token probability | 0.62 (0.60 - 0.64) vs 0.74 (0.72 - 0.75) | <.001 |
|  | Entropy vs Expressed confidence | 0.74 (0.72 - 0.76) vs 0.60 (0.57 - 0.62) | <.001 |
|  | Entropy vs Perplexity | 0.74 (0.72 - 0.76) vs 0.62 (0.60 - 0.64) | <.001 |
|  | Entropy vs Token probability | 0.74 (0.72 - 0.76) vs 0.74 (0.72 - 0.75) | .23 |
| **Gemma2-27b** | Expressed confidence vs Perplexity | 0.56 (0.54 - 0.58) vs 0.65 (0.62 - 0.67) | <.001 |
|  | Perplexity vs Token probability | 0.65 (0.62 - 0.67) vs 0.76 (0.74 - 0.78) | <.001 |
|  | Entropy vs Expressed confidence | 0.77 (0.75 - 0.78) vs 0.56 (0.54 - 0.58) | <.001 |
|  | Entropy vs Perplexity | 0.77 (0.75 - 0.78) vs 0.65 (0.62 - 0.67) | <.001 |
|  | Entropy vs Token probability | 0.77 (0.75 - 0.78) vs 0.76 (0.74 - 0.78) | <.001 |

**Table S19.** Bibliography summary

| **First Author** | **Journal** | **Date** | **Metrics used** | **Result** | **Models** | **Dataset** | **Language** | **Question types** |
| --- | --- | --- | --- | --- | --- | --- | --- | --- |
| Kufel et al. | Polish Journal of Radiology | 2023 | - Expressed confidence | - Significant discriminatory capability of expressed confidence | GPT3.5 only | Cutsom 120 radiology questions | Polish only | Single choice |
| Schubert et al. | JAMA Neurology | 2023 | - Expressed confidence | - Significant but low discriminatory capability of expressed confidence - Tendency toward overconfidence | GPT3.5 and GPT4 | 2036 publicly available neurology board questions | English only | Single choice |
| Liévin et al. | Patterns | 2024 | - Multiple sampling | - Good calibration of multiple sampling | GPT3, Llama 2, Vicuna, Guanaco, Falcon, MPT, and GPT-NeoX | MedQA, MedMCQA and PubMedQA | English only | Single choice |
| Rydzewski et al. | NEJM AI | 2024 | - Expressed confidence - Multiple sampling | - Significant discriminatory capability of both expressed confidence and multiple sampling - Tendency towards overconfidence for expressed confidence - Fixed false beliefs with multiple sampling - No comparison between methods | GPT-4 Turbo, Gemini 1.0 Ultra, Mixtral 8x7B and LLaMA 2 | Mix of publicly available and custom 2044 oncology questions | English only | Unknown |
| Krishna et al. | Radiology | 2024 | - Expressed confidence | - Overconfidence for expressed confidence - Better results with more capable model | GPT3.5 and GPT4 | Custom 150 radiology board–style multiple-choice text-based questions | English only | Open-ended questions |
| Wada et al. | Diagnostics | 2024 | - Expressed confidence | - Significant discriminatory capability of expressed confidence | GPT-4 Turbo only | 751 publicly available radiology questions | English only | Open-ended questions |
| Farquhar et al. | Nature | 2024 | - Semantic entropy using multiple sampling | - Significant discriminatory capability of semantic entropy | Llama 2, Falcon and Mistral | Publicly available BioASQ, SQuAD, TriviaQA, SVAMP and NQ-Open | English only | Open-ended questions |
| Levine et al. | The Lancet DIgital Heatlth | 2024 | - Multiple sampling | - Good calibration of multiple sampling | GPT-3 only | Custom 48 clinical vignettes | English only | Open-ended questions |
| Savage et al. | Journal of the American Medical Informatics Association | 2024 | - Expressed confidence - Multiple sampling - Token level probabilities | - Significant discriminatory capability of both expressed confidence, token level probabilities and multiple sampling - Significantly higher performance of multiple sampling as compared to other methods | GPT3.5, GPT4, Llama 2 70B, Llama 3 70B | Mix of 618 publicly available and 105 custom questions | English only | Open-ended questions |
