# Supplementary material for: Token Probabilities to Mitigate Large Language Models Overconfidence in Answering Medical Questions: Quantitative Study
Source: J Med Internet Res. 2025 Aug 29;27:e64348. doi: 10.2196/64348 (PMC12396779; doi:10.2196/64348)
Supplement: Multimedia Appendix 2 [file jmir-v27-e64348-s002.docx]

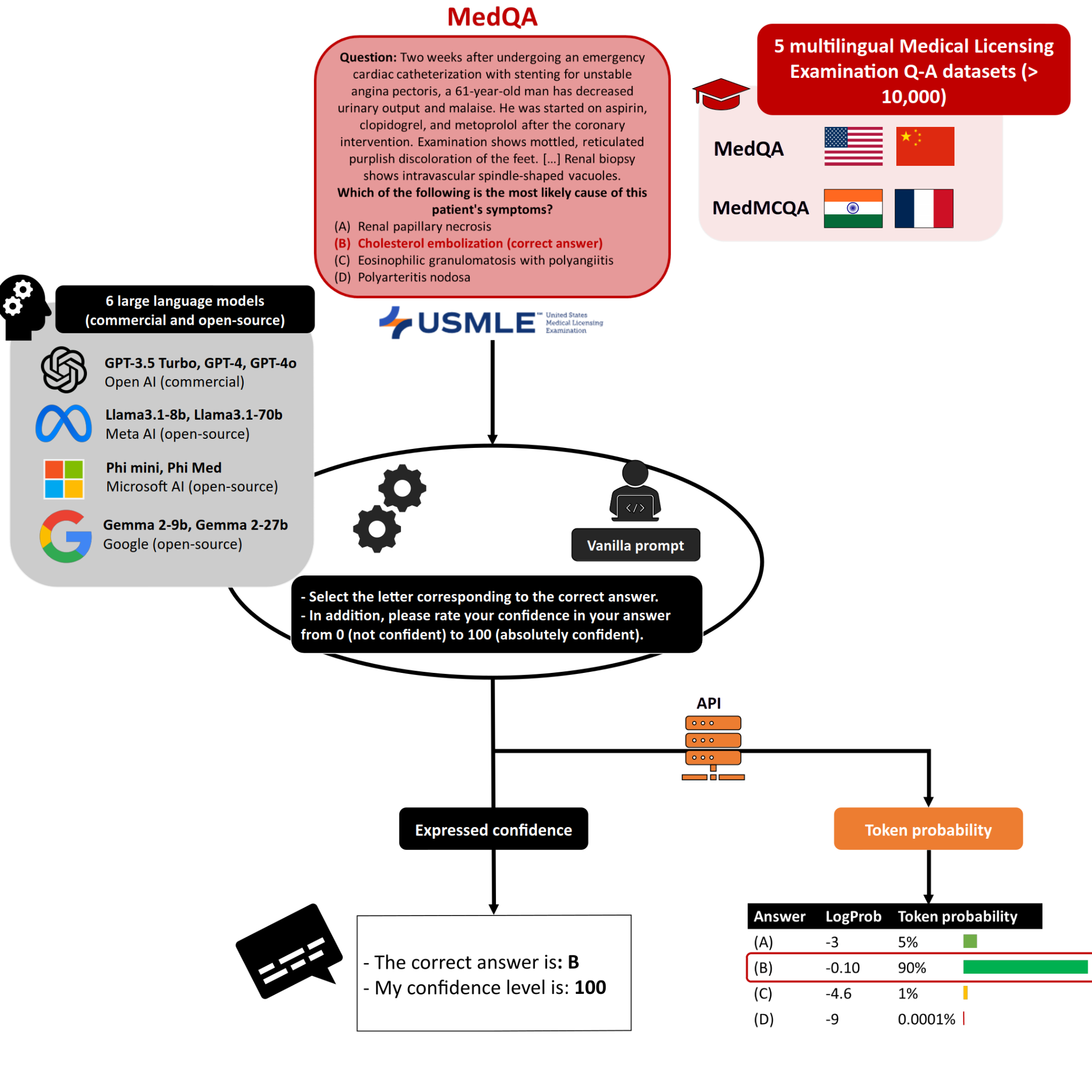


**Figure S1.** Expressed confidence and response token probability are extracted from Chatbots’ responses.


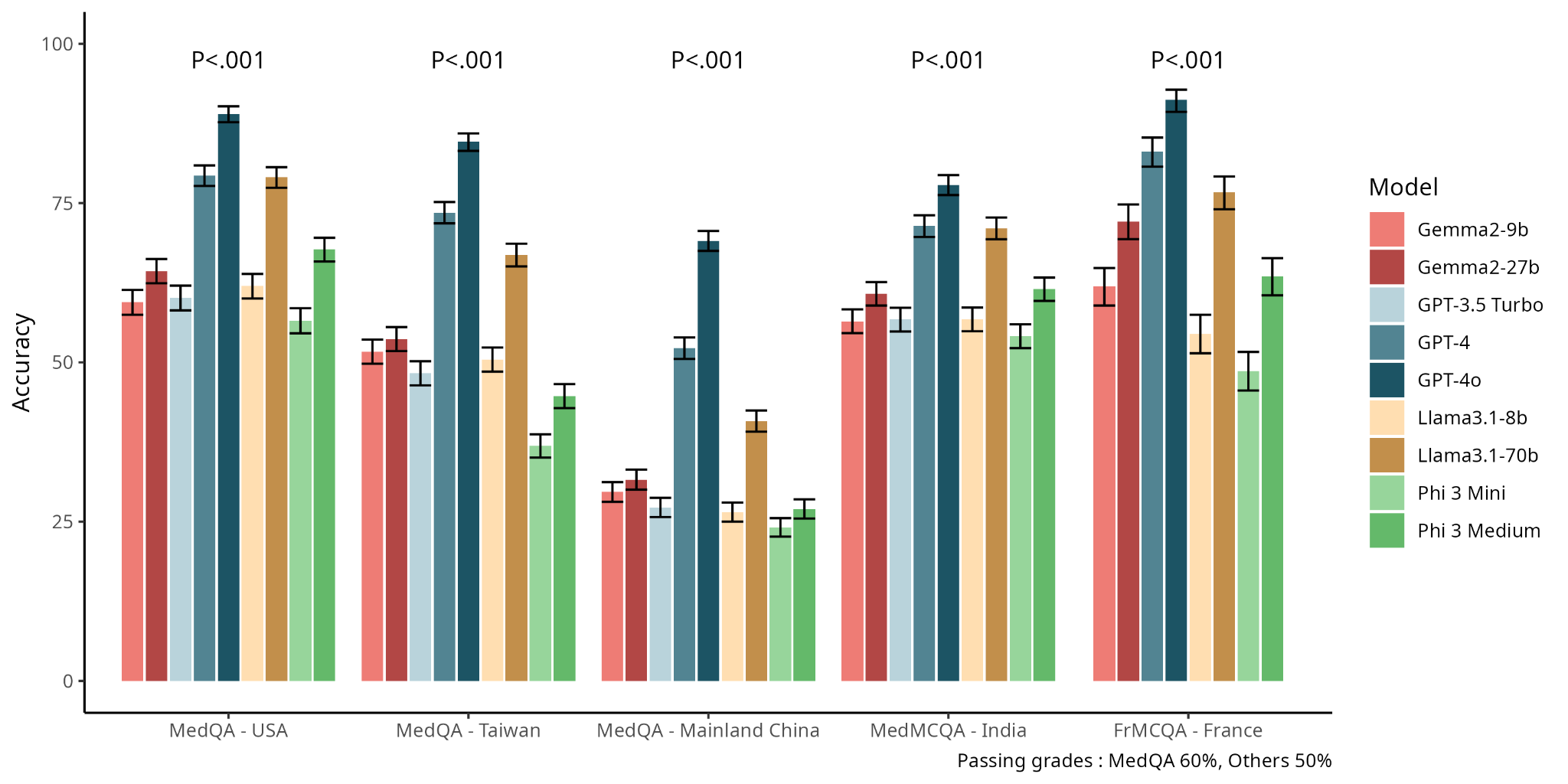


**Figure S2.** Accuracy of each LLM across five multilingual medical question-answer datasets.


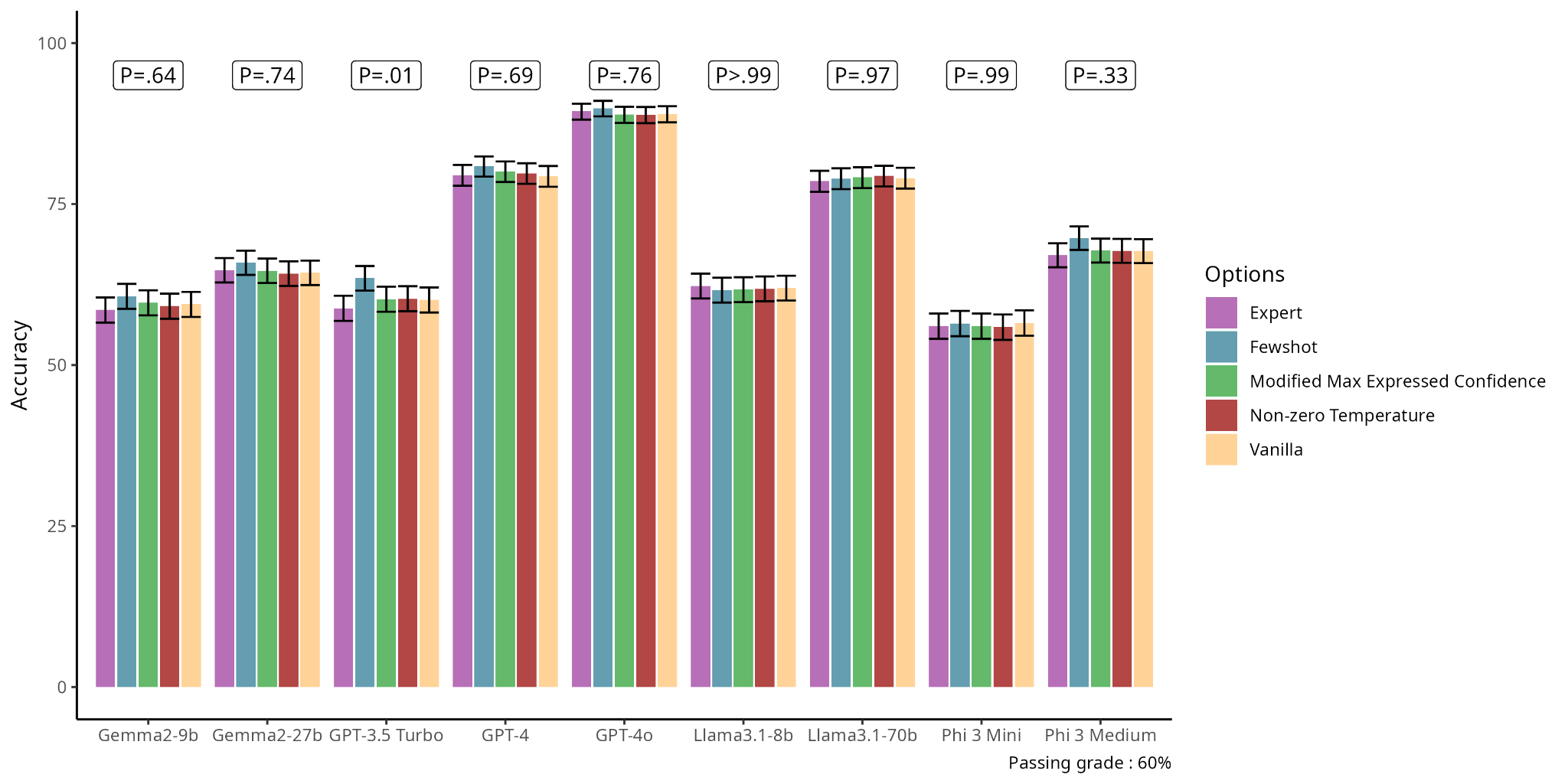


**Figure S3.** Effect of prompting method on the accuracy of each LLM in the US MedQA dataset (n = 2,487).


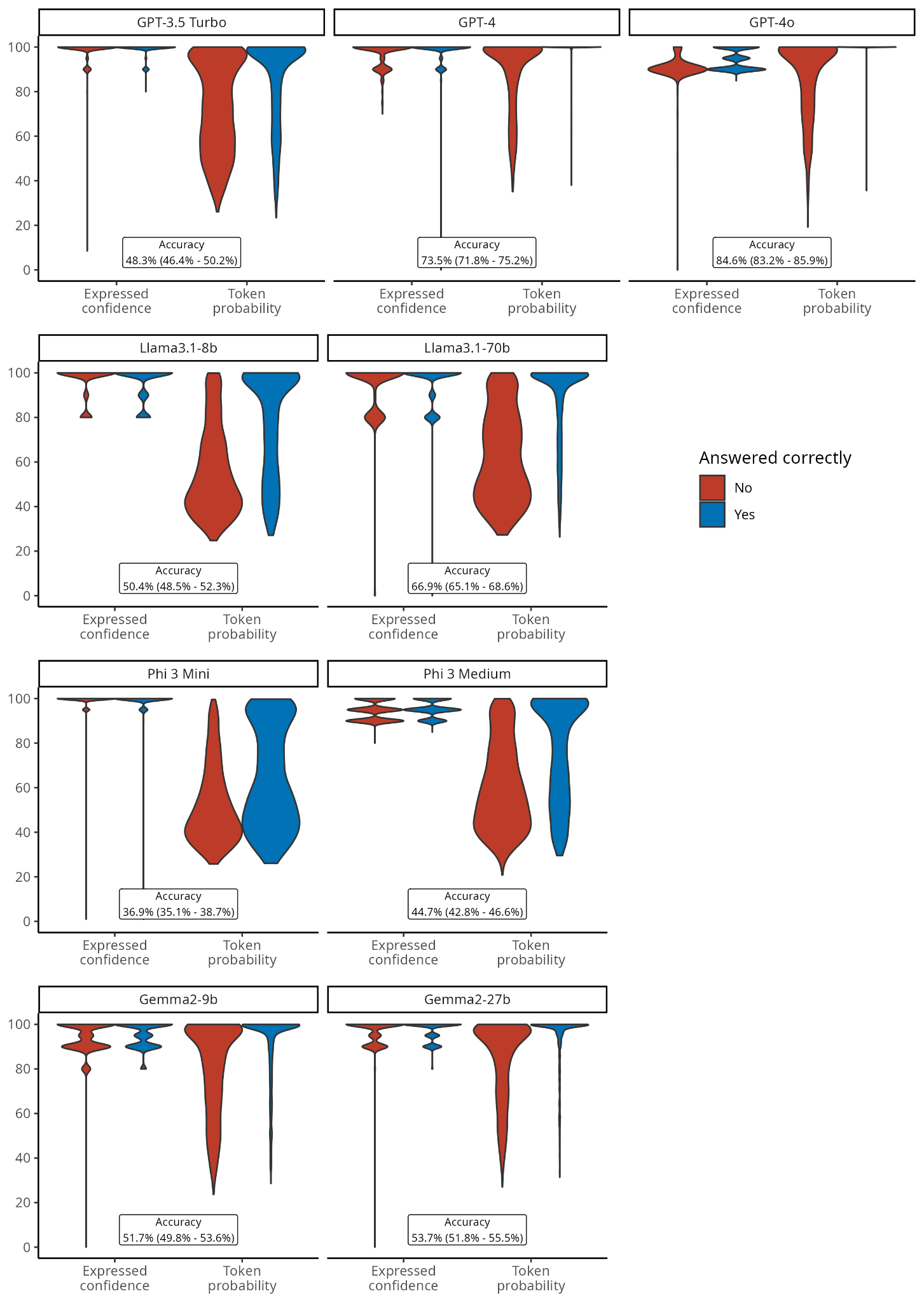


**Figure S4.** Response token probability vs. expressed confidence of the LLMs (Taiwan MedQA - n = 2,734 questions)


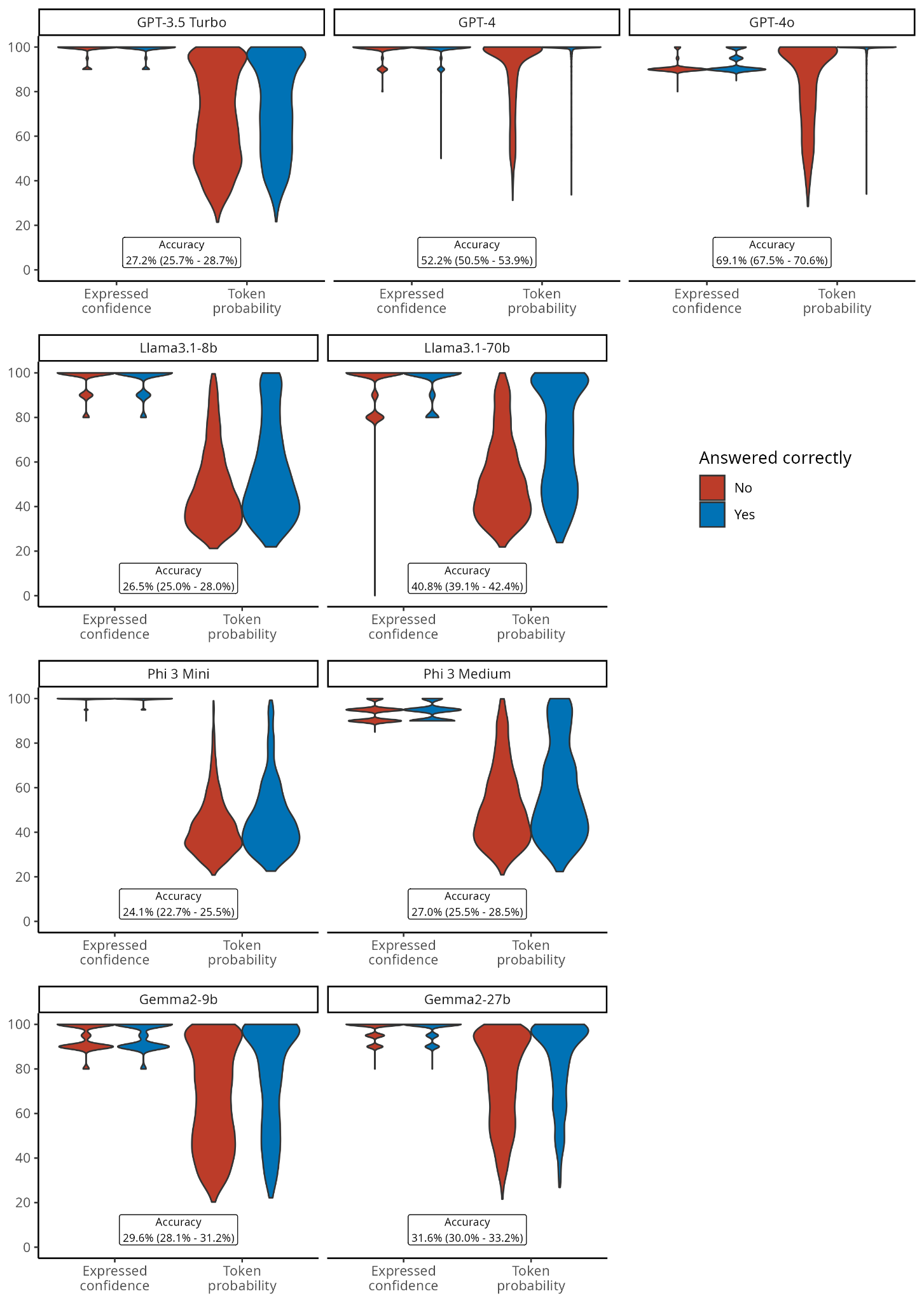


**Figure S5.** Response token probability vs. expressed confidence of the LLMs (Mainland China MedQA - n = 3,414 questions)


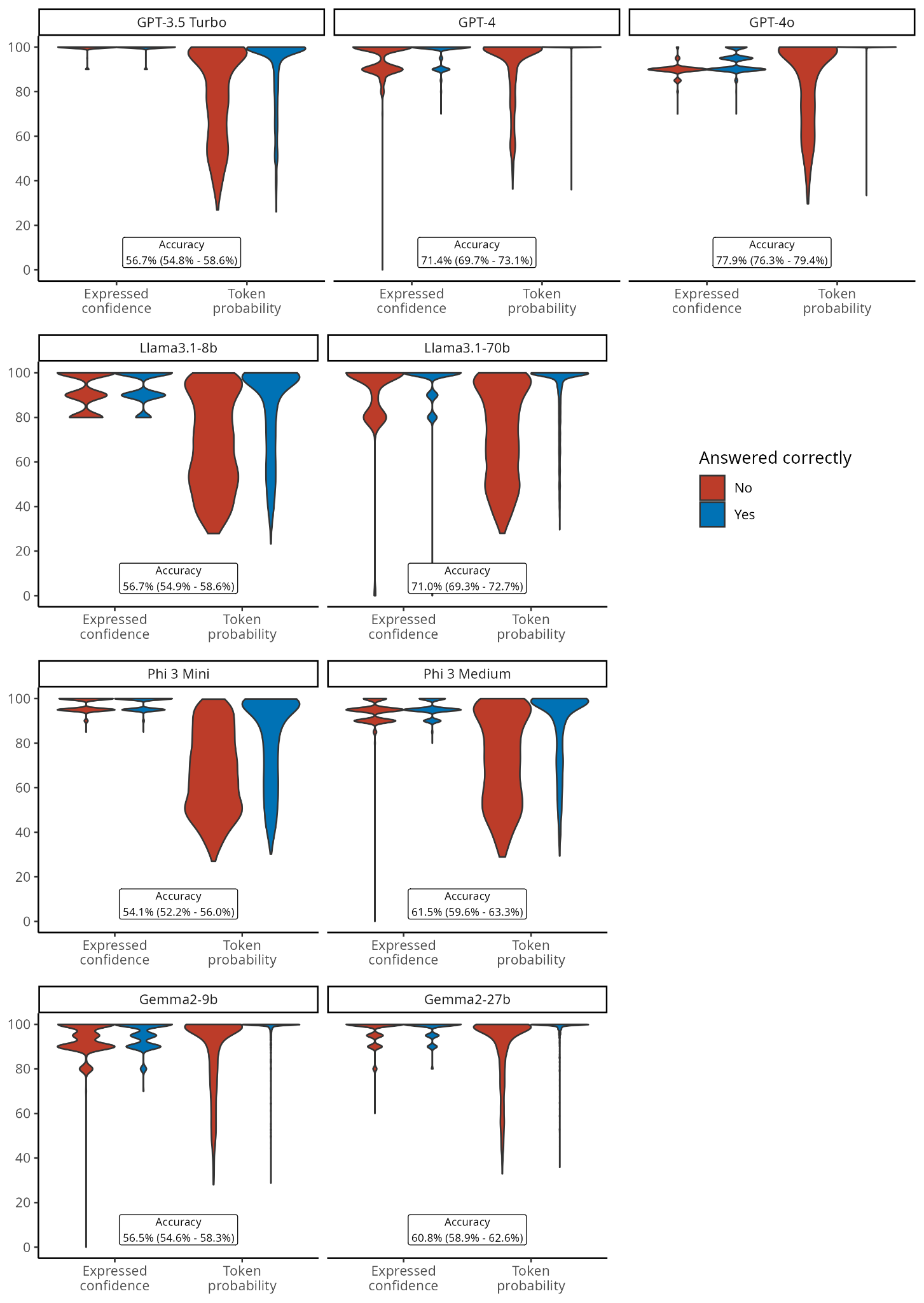


**Figure S6.** Response token probability vs. expressed confidence of the LLMs (India MedMCQA - n = 2,763 questions)


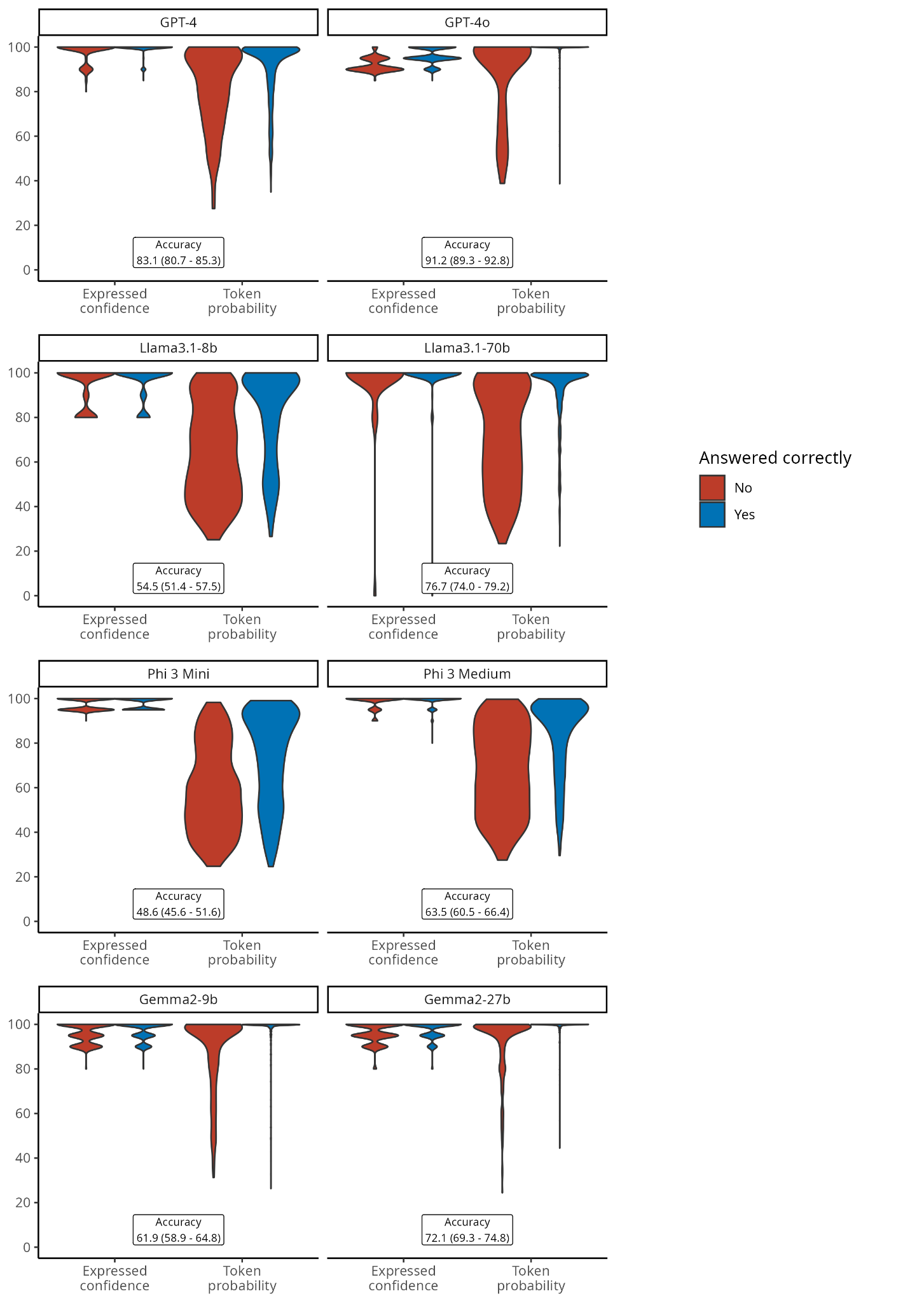


*GPT-3.5 Turbo was excluded because of too many uninterpretable answers*

**Figure S7.** Response token probability vs. expressed confidence of the LLMs (French FrMedMCQA dataset - n = 1,076 questions)


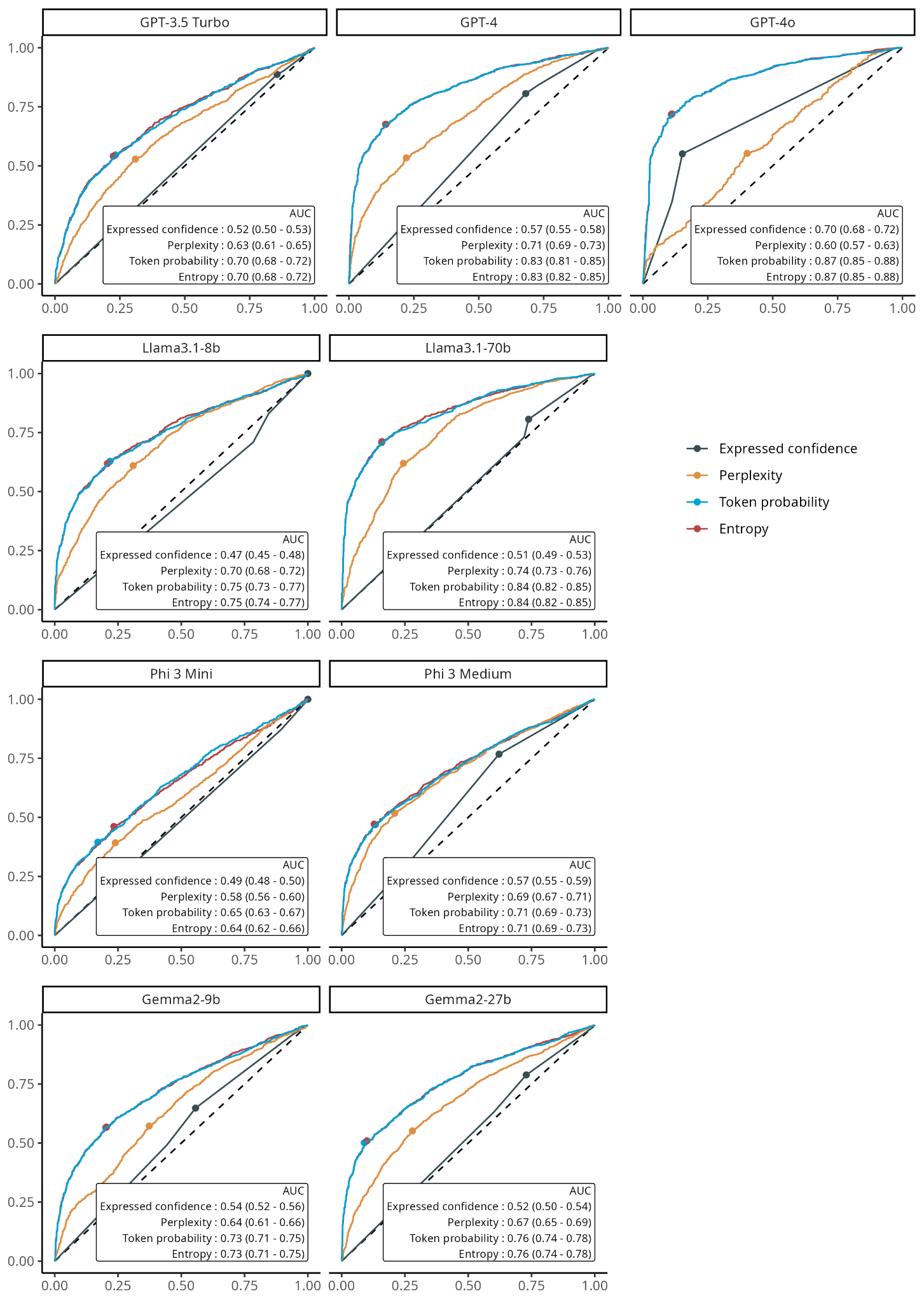


**Figure S8.** Receiver Operating Characteristic curve and discriminative power of 4 uncertainty metrics in predicting answer accuracy of LLMs (Taiwan MedQA - n = 2,734 questions)


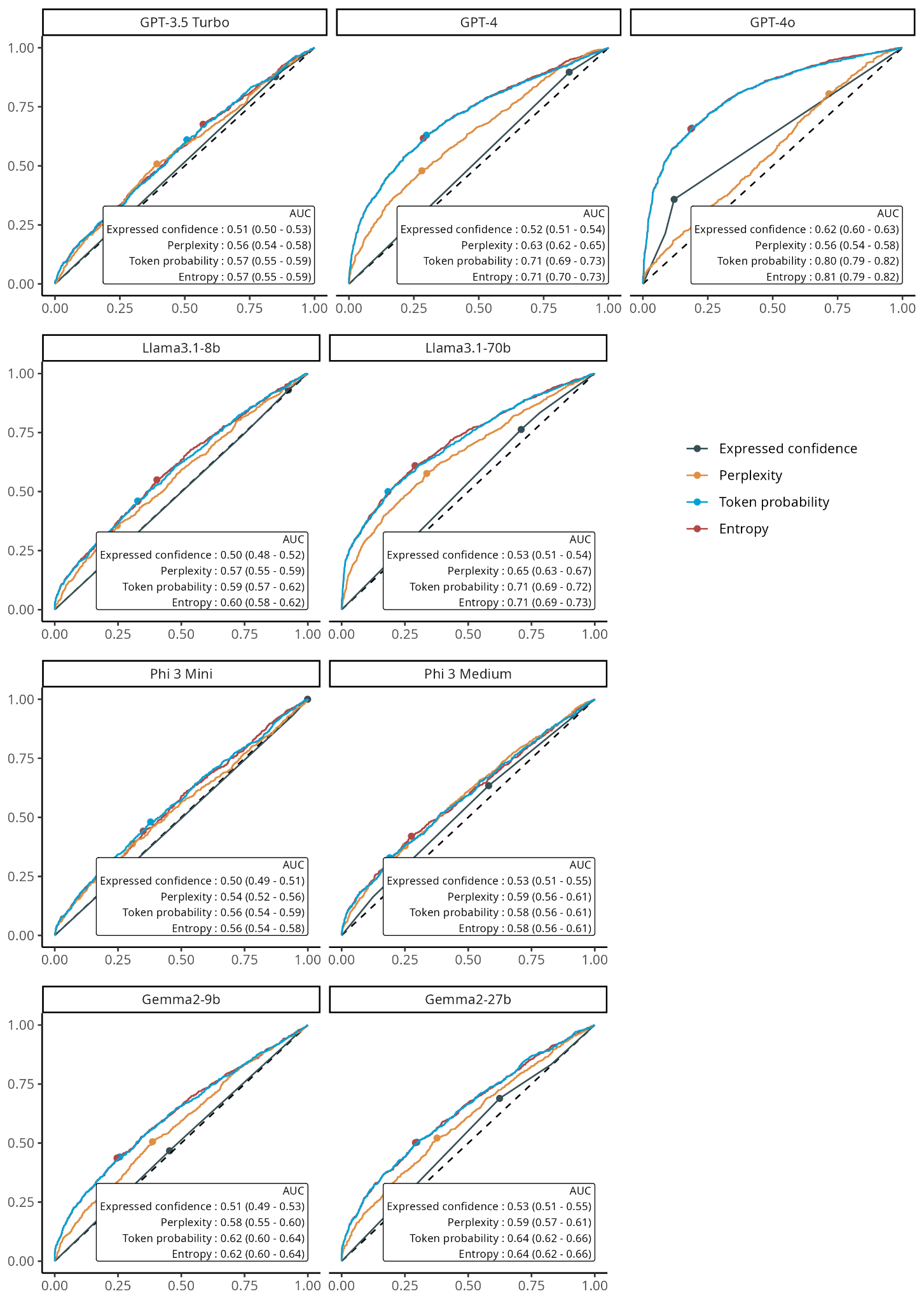


**Figure S9.** Receiver Operating Characteristic curve and discriminative power of 4 uncertainty metrics in predicting answer accuracy of LLMs (Mainland China MedQA - n = 3,414 questions)


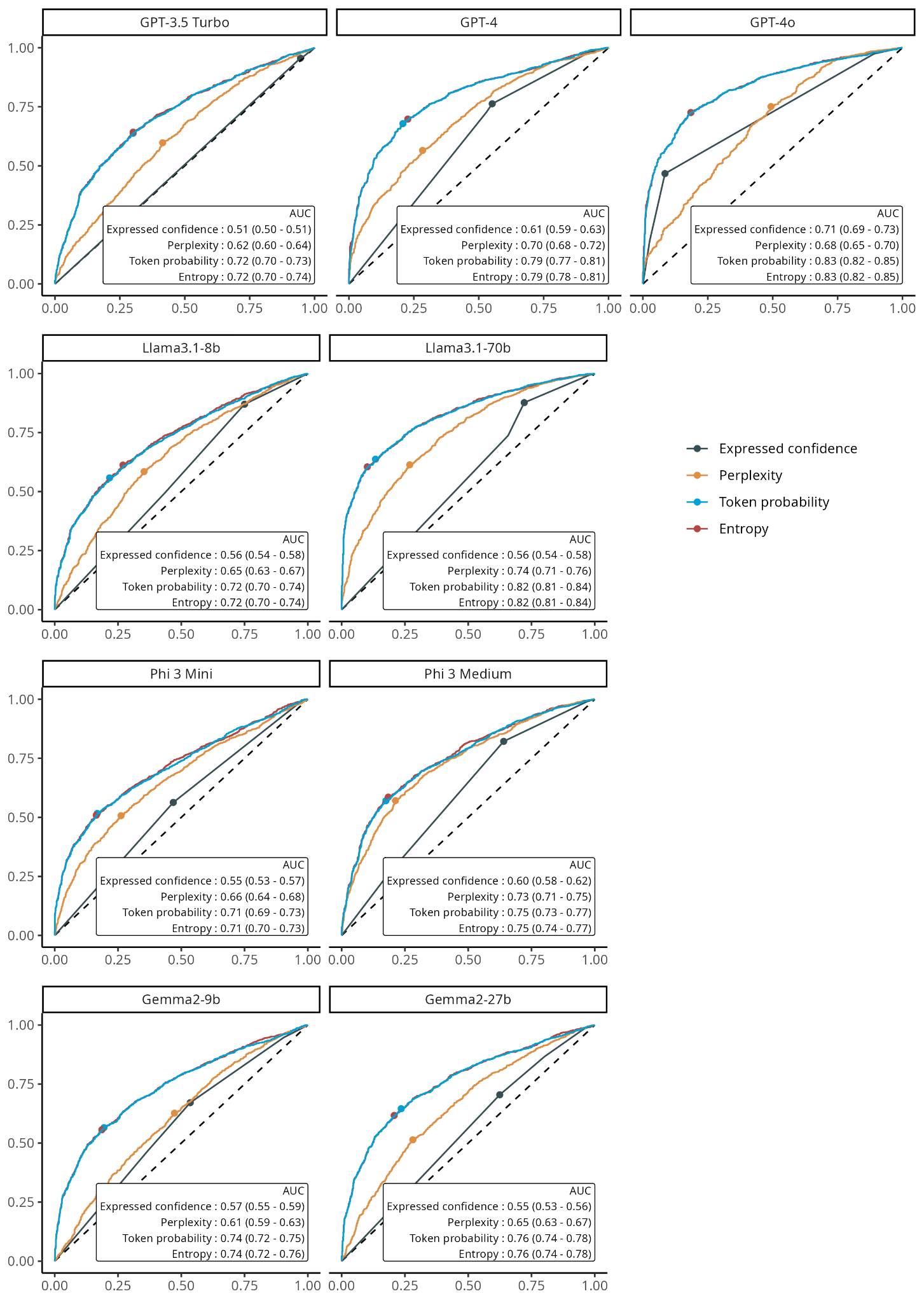


**Figure S10.** Receiver Operating Characteristic curve and discriminative power of 4 uncertainty metrics in predicting answer accuracy of LLMs (India MedMCQA - n = 2,763 questions)


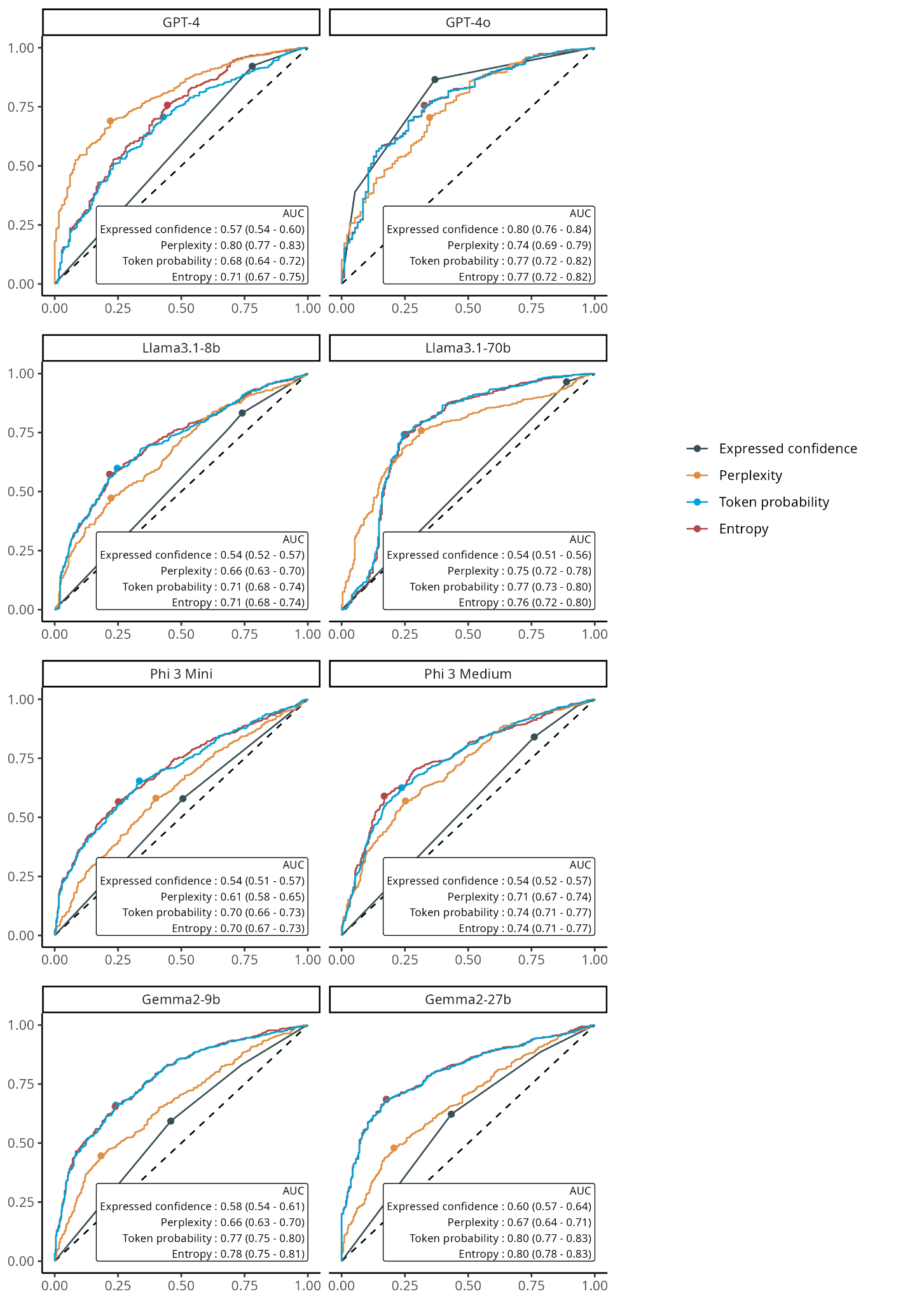


*GPT-3.5 Turbo was excluded because of too many uninterpretable answers*

**Figure S11.** Receiver Operating Characteristic curve and discriminative power of 4 uncertainty metrics in predicting answer accuracy of LLMs (French FrMedMCQA - n = 1,076 questions)


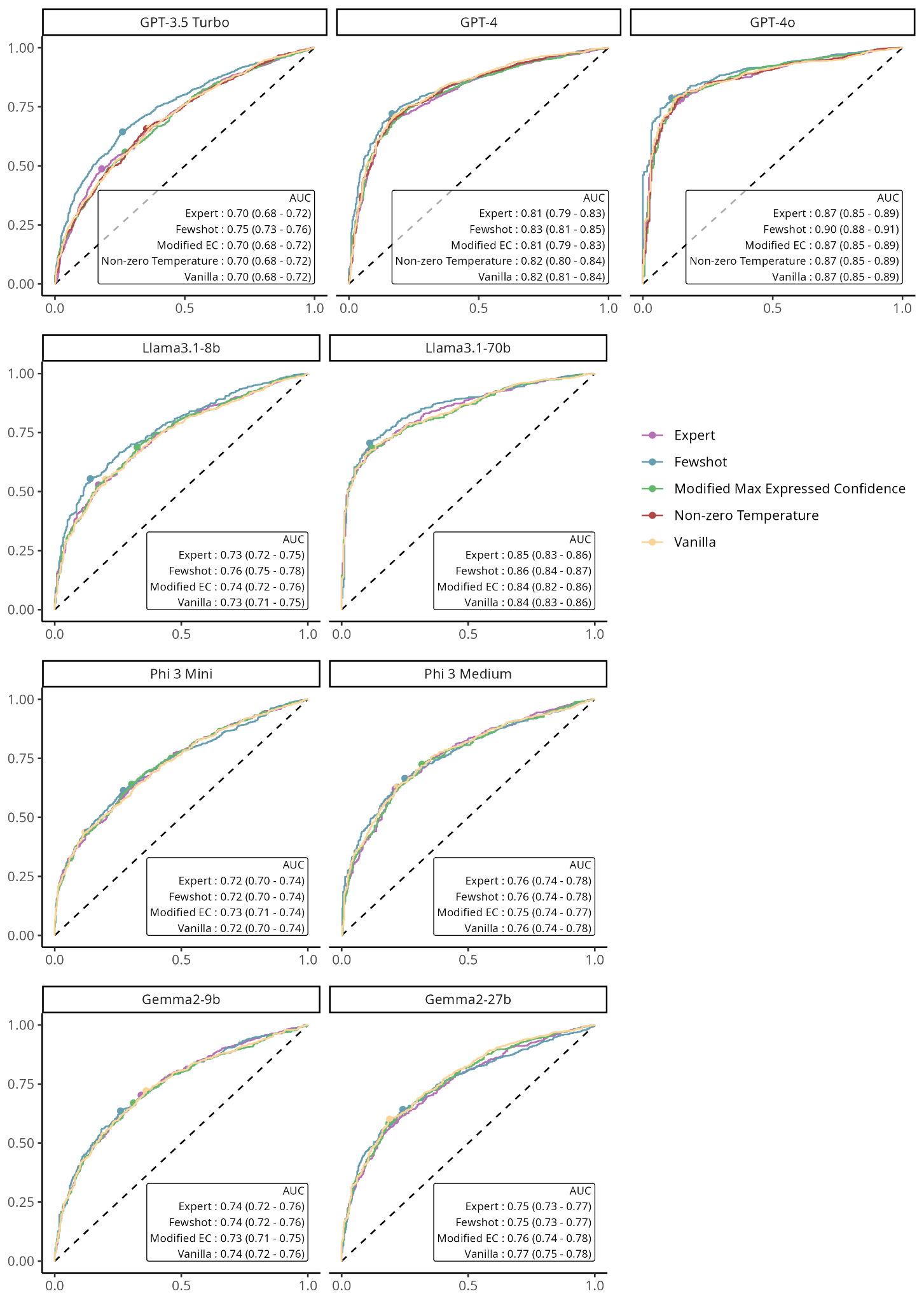


**Figure S12**. Effect of prompting techniques on Receiver Operating Characteristic curve and discriminative power of the response tokens’s Shannon entropy in predicting answer accuracy of LLMs (US MedQA dataset - n = 2,487 questions)


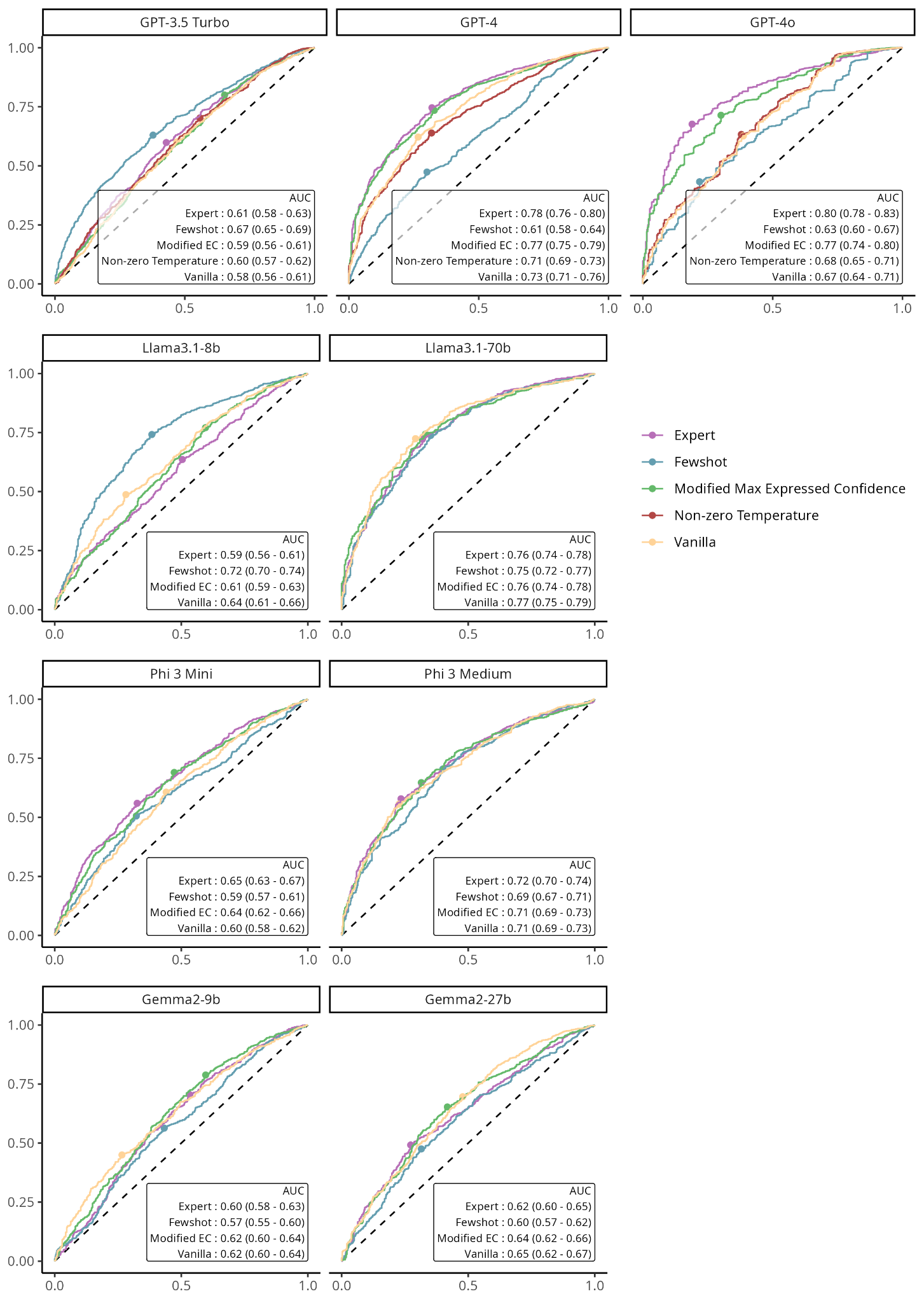


**Figure S13**. Effect of prompting techniques on Receiver Operating Characteristic curve and discriminative power of the response message’s perplexity in predicting answer accuracy of LLMs (US MedQA dataset - n = 2,487 questions)


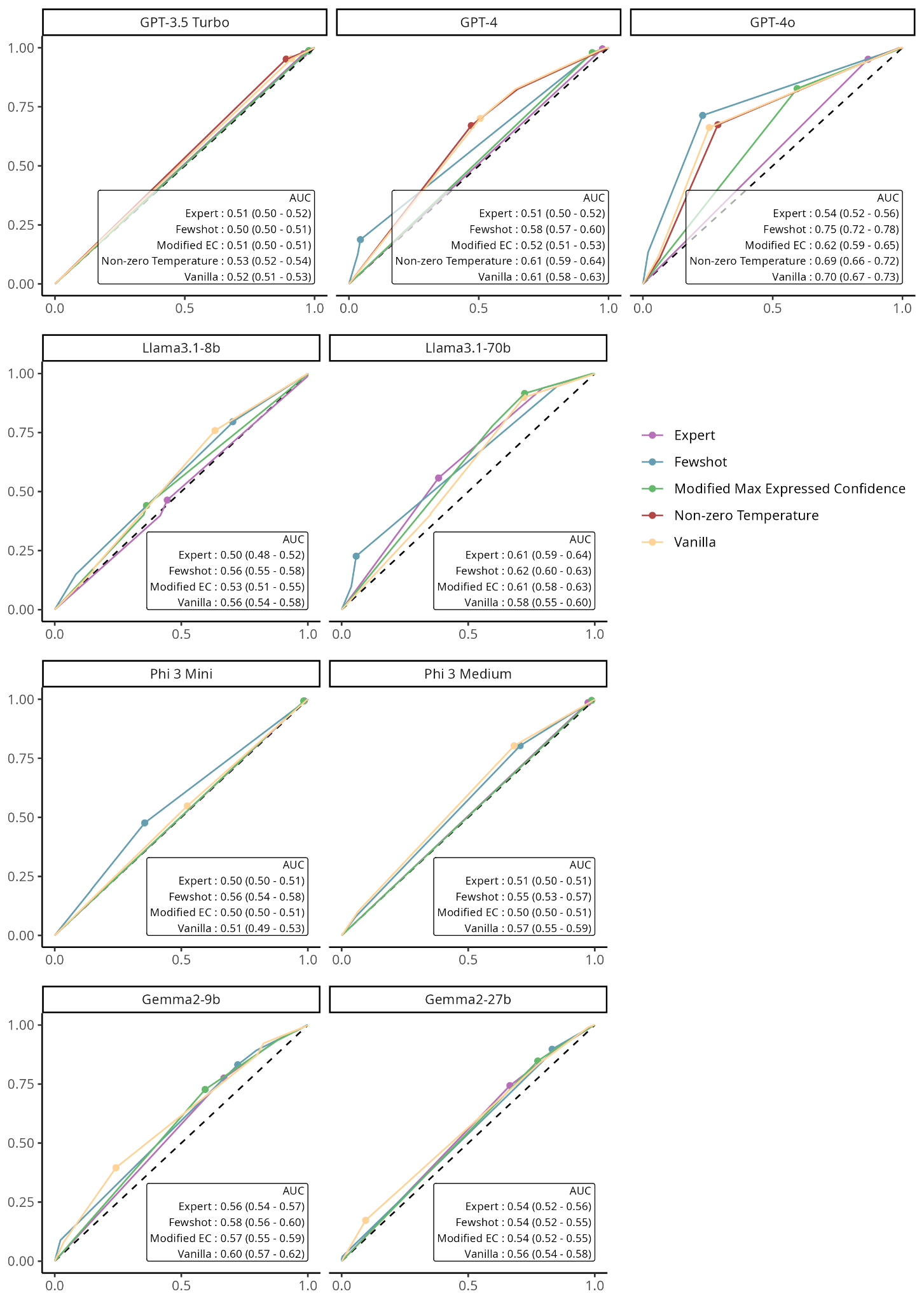


**Figure S14**. Effect of prompting techniques on Receiver Operating Characteristic curve and discriminative power of the model’s expressed confidence in predicting answer accuracy of LLMs (US MedQA dataset - n = 2,487 questions)


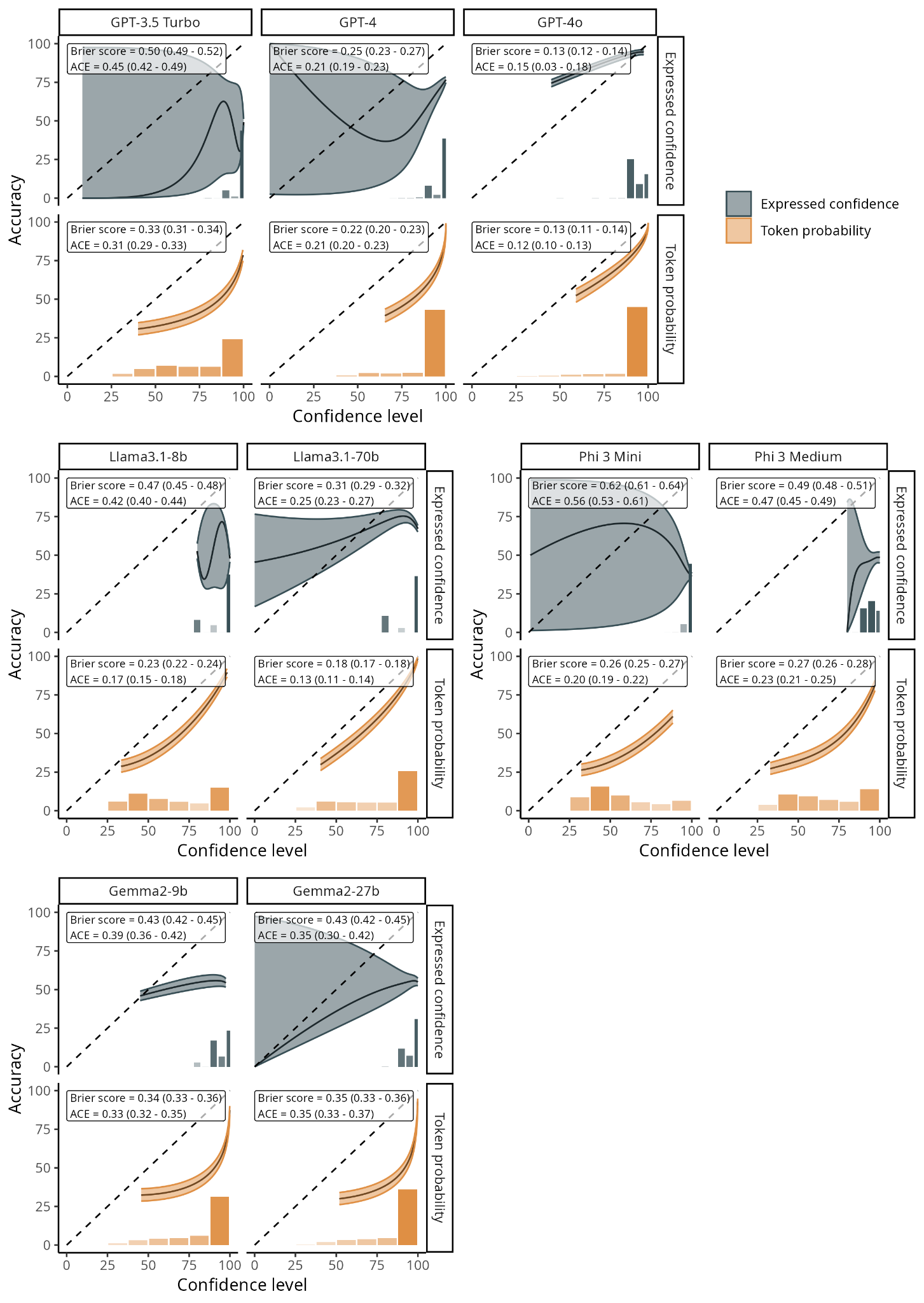


**Figure S15.** Calibration plots comparing expressed confidence vs. Response token probability in predicting answer accuracy of LLMs (Taiwan MedQA - n = 2,734 questions)


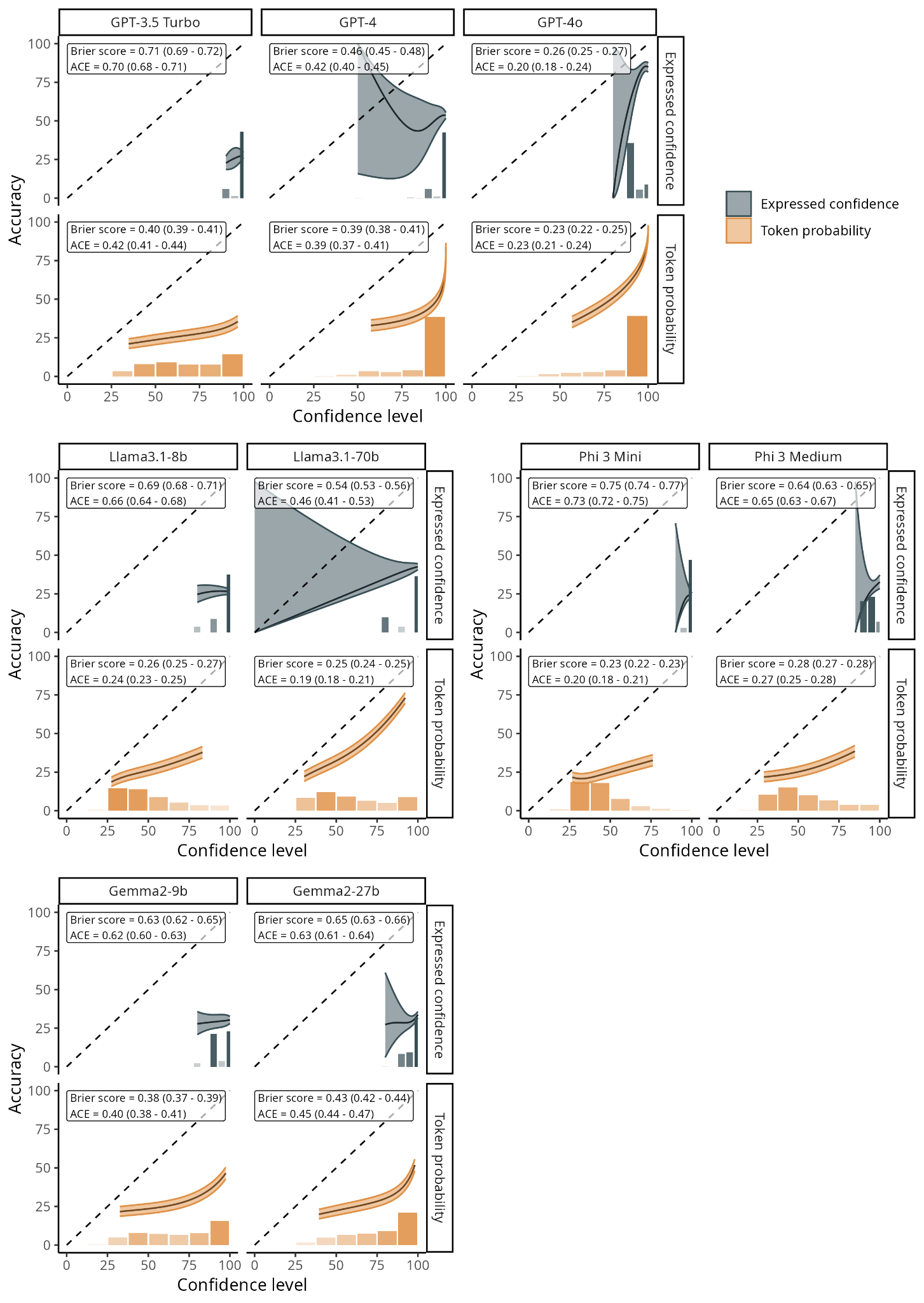


**Figure S16.** Calibration plots comparing expressed confidence vs. response token probability in predicting answer accuracy of LLMs (Mainland China MedQA - n = 3,414 questions)


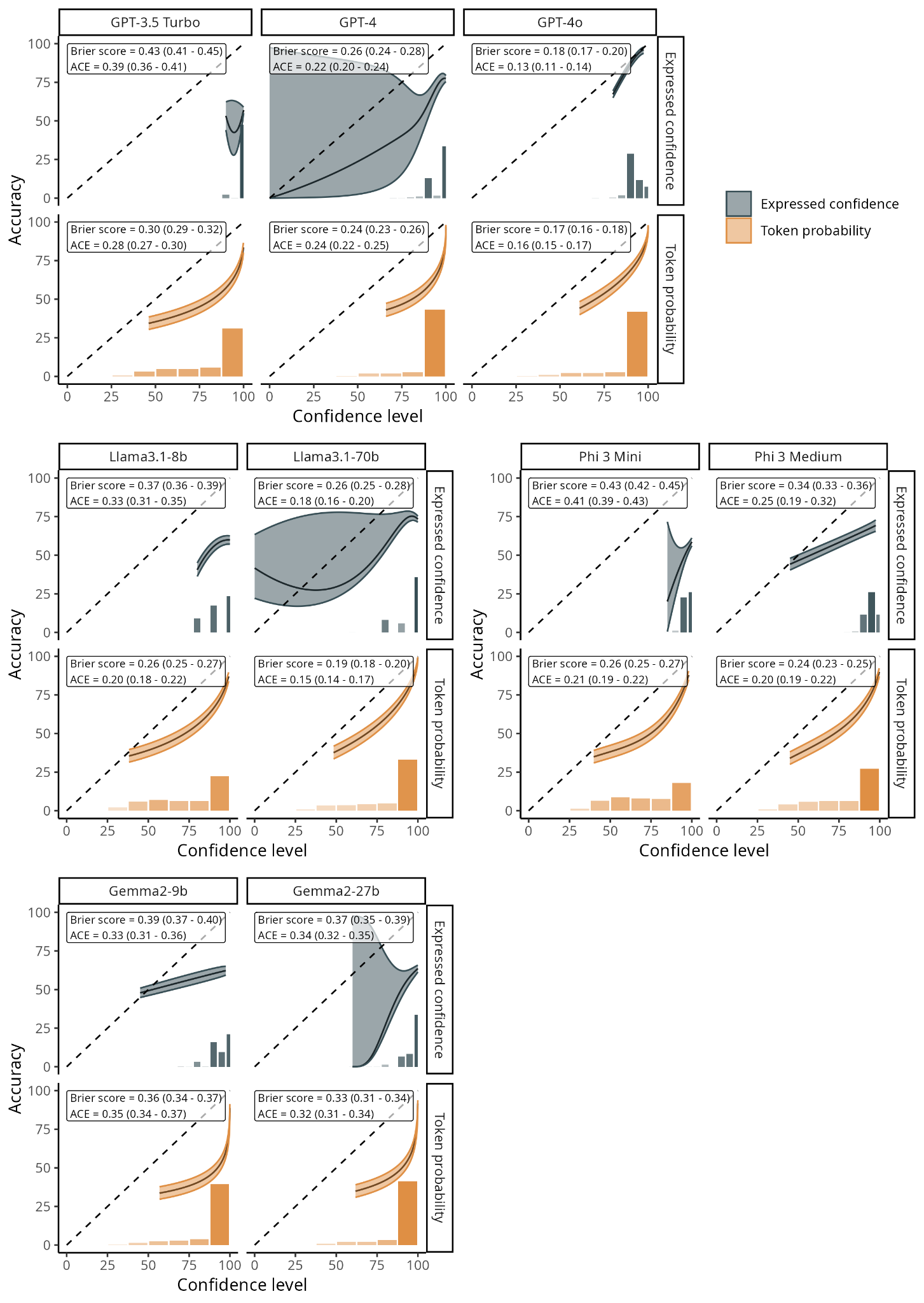


**Figure S17.** Calibration plots comparing expressed confidence vs. response token probability in predicting answer accuracy of LLMs (India MedMCQA - n = 2,763 questions)


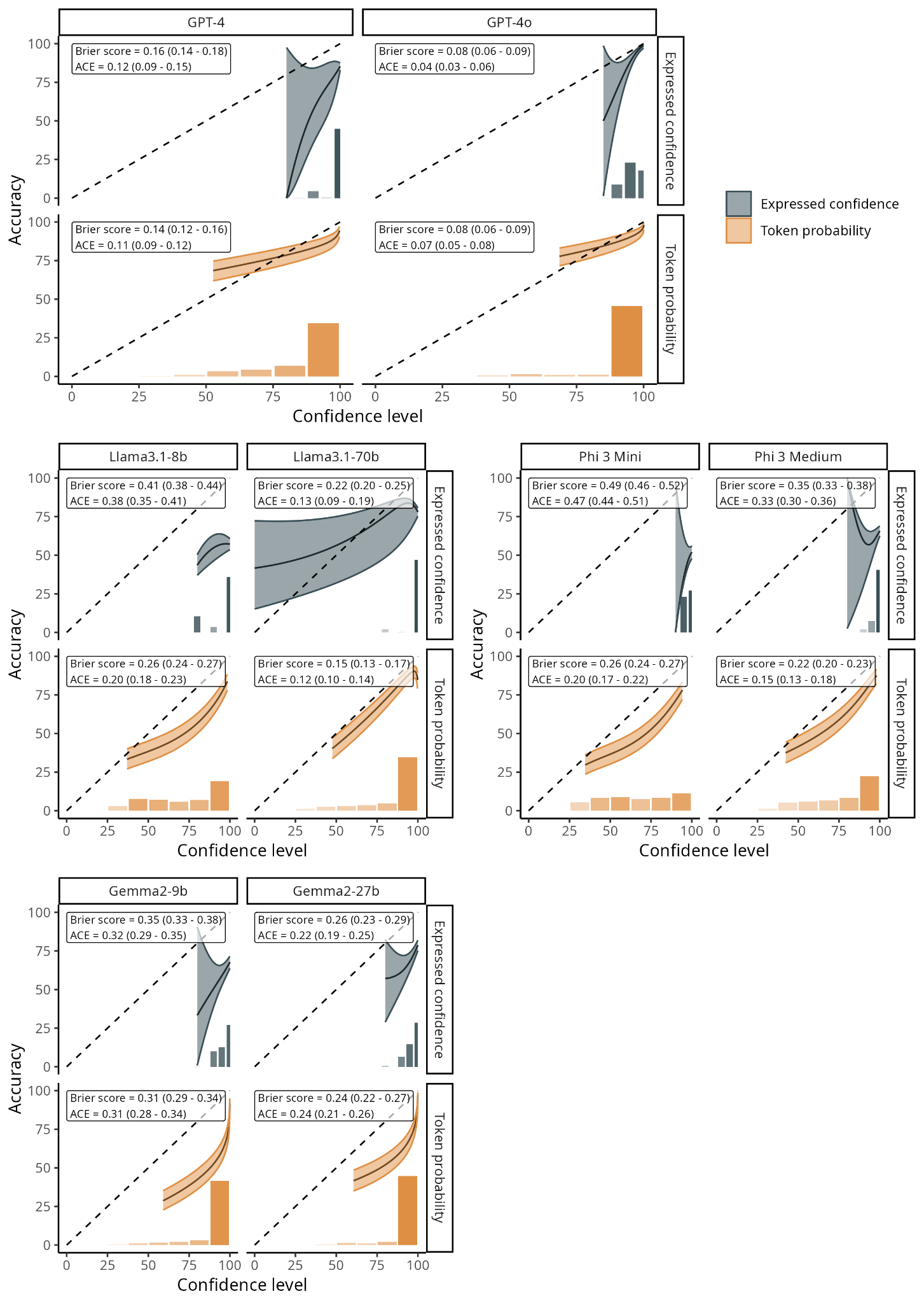


**Figure S18.** Calibration plots comparing expressed confidence vs. response token probability in predicting answer accuracy of LLMs (French FrMedMCQA - n = 1,076 questions)


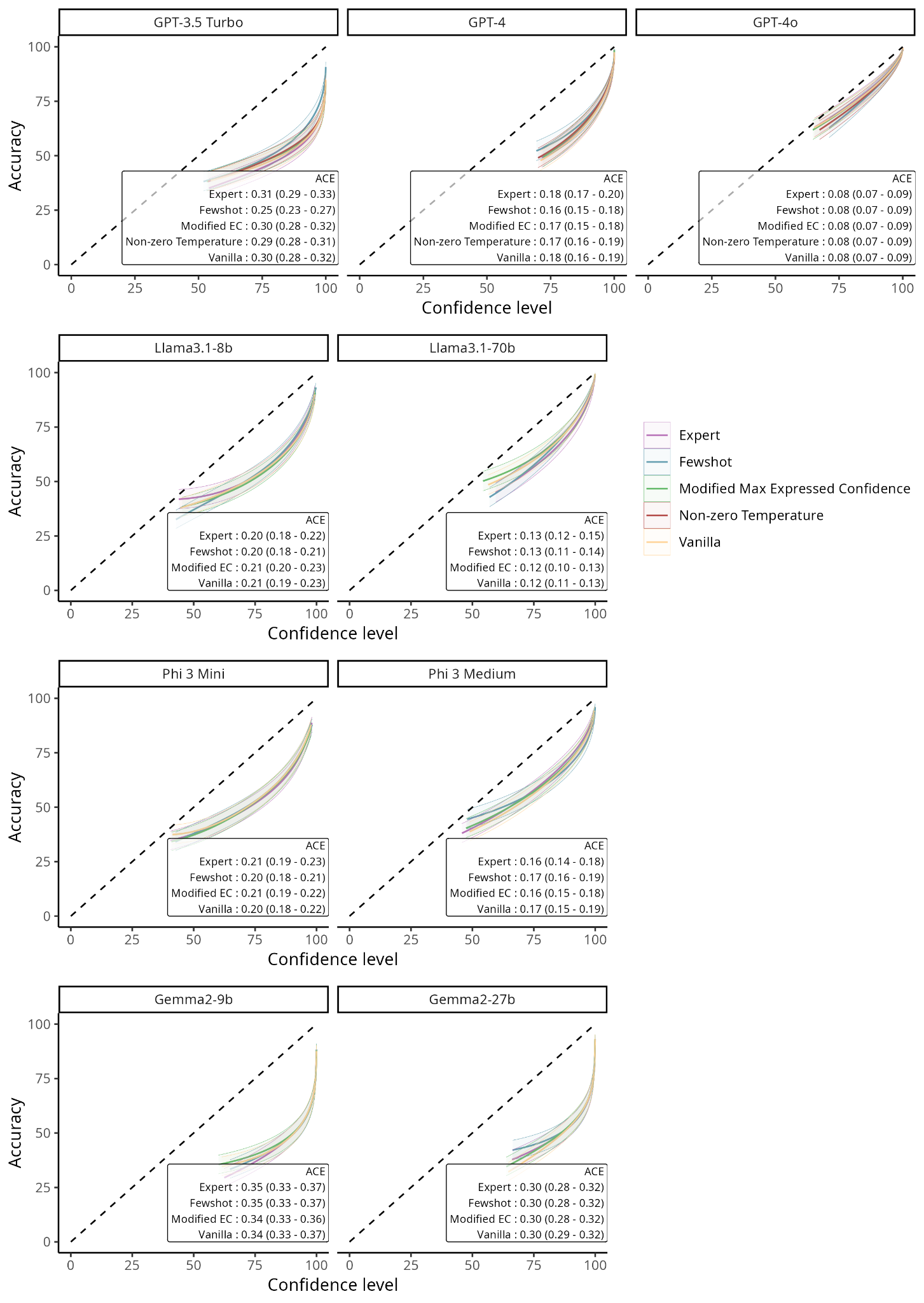


**Figure S19.** Effect of prompting method on the calibration of the token probability in predicting answer accuracy of LLMs (US MedQA dataset - n = 2,487 questions)


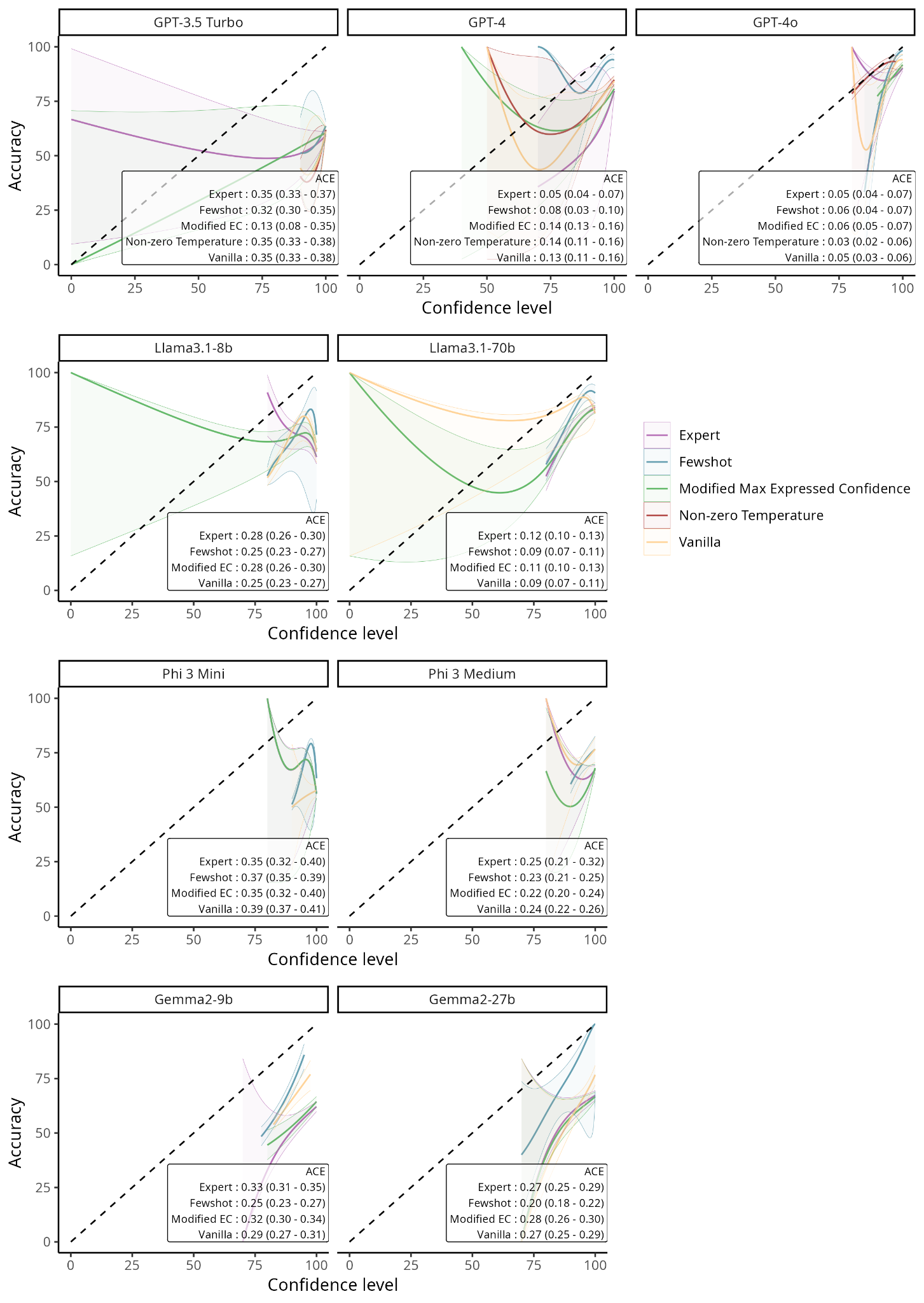


**Figure S20.** Effect of prompting method on the calibration of the expressed confidence in predicting answer accuracy of LLMs (US MedQA dataset - n = 2,487 questions)
